# Autism risk gene POGZ promotes chromatin accessibility and expression of clustered synaptic genes

**DOI:** 10.1016/j.celrep.2021.110089

**Published:** 2021-12-07

**Authors:** Eirene Markenscoff-Papadimitriou, Fadya Binyameen, Sean Whalen, James Price, Kenneth Lim, Athena R. Ypsilanti, Rinaldo Catta-Preta, Emily Ling-Lin Pai, Xin Mu, Duan Xu, Katherine S. Pollard, Alex S. Nord, Matthew W. State, John L. Rubenstein

**Affiliations:** 1 Department of Psychiatry, Langley Porter Psychiatric Institute, UCSF Weill Institute for Neurosciences, University of California, San Francisco, CA, USA; 2 Gladstone Institutes, San Francisco, CA, USA; 3 Departments of Neurobiology, Physiology, and Behavior and Psychiatry and Behavioral Sciences, University of California, Davis, Davis, CA, USA; 4 Department of Radiology; 5 Chan-Zuckerberg Biohub, San Francisco, CA, USA; 6 Institute for Computational Health Sciences, University of California, San Francisco, CA, USA; 7 Institute for Human Genetics, University of California, San Francisco, CA, USA; 8 Department of Epidemiology and Biostatistics, University of California, San Francisco, CA, USA; 9 Quantitative Biology Institute, University of California, San Francisco, CA, USA; 10 Lead contact

## Abstract

Deleterious genetic variants in *POGZ*, which encodes the chromatin regulator *Pogo* Transposable Element with ZNF Domain protein, are strongly associated with autism spectrum disorder (ASD). Although it is a high-confidence ASD risk gene, the neurodevelopmental functions of POGZ remain unclear. Here we reveal the genomic binding of POGZ in the developing forebrain at euchromatic loci and gene regulatory elements (REs).We profile chromatin accessibility and gene expression in *Pogz*^−/−^ mice and show that POGZ promotes the active chromatin state and transcription of clustered synaptic genes. We further demonstrate that POGZ forms a nuclear complex and co-occupies loci with ADNP, another high-confidence ASD risk gene, and provide evidence that POGZ regulates other neurodevelopmental disorder risk genes as well. Our results reveal a neurodevelopmental function of an ASD risk gene and identify molecular targets that may elucidate its function in ASD.

## In brief

Markenscoff-Papadimitriou et al. map POGZ occupancy in developing human and mouse brain tissue and find POGZ binds euchromatic loci at promoters and enhancers. Analyses of *Pogz*^−/−^ mice reveal POGZ specifically promotes the chromatin accessibility and expression of clustered synapse genes, and biochemical analyses show POGZ co-occupies loci with ADNP.

## Graphical Abstract

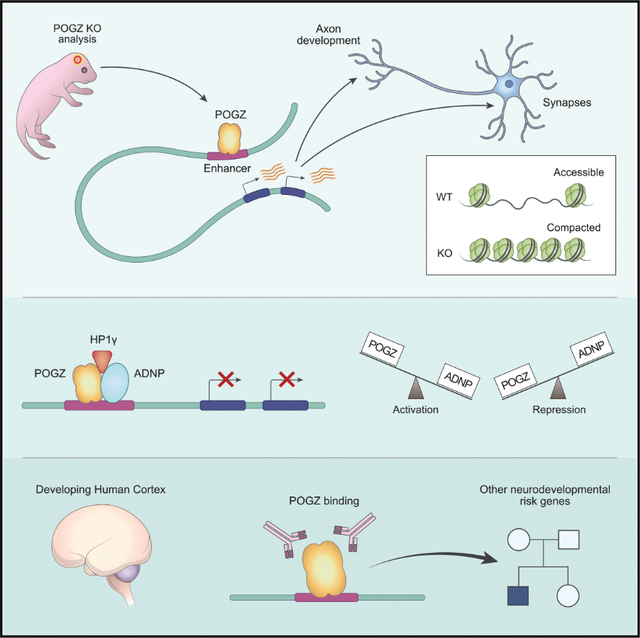

## INTRODUCTION

Chromatin packaging of DNA is a dynamic process that determines the transcriptional potential of a cell. Chromatin regulators compartmentalize the nucleus into domains of euchromatin and heterochromatin: euchromatin is characterized by accessible DNA and is associated with transcription, while heterochromatin is characterized by compacted DNA and is associated with transcriptional repression. Deleterious genetic variants in chromatin regulator genes are strongly linked to neurodevelopmental disorders (NDDs) including autism spectrum disorder (ASD) and developmental delay (Satterstrom et al., 2020; Sanders et al., 2015; De Rubeis et al., 2014; Deciphering Developmental Disorders Study, 2015). Understanding how these chromatin regulators impact gene regulation during brain development may illuminate the underlying mechanisms contributing to social and intellectual disability and potentially other neuropsychiatric syndromes (Gompers et al., 2017; Jung et al., 2017; Cappi et al., 2020).

POGZ has been identified as a high confidence ASD risk gene in whole exome sequencing studies of patient cohorts (Satterstrom et al., 2020; Sanders et al., 2015; De Rubeis et al., 2014; Stessman et al., 2016; Iossifov et al., 2014). Subsequent to its association with idiopathic ASD, “genotype-first” analyses led to the characterization of White-Sutton syndrome, defined by pathogenic variants in POGZ. This syndrome is marked by distinctive facial features along with intellectual disability (ID), ASD, and neurological and gastrointestinal abnormalities (Assia Batzir et al., 2020).

POGZ encodes a protein with a N-terminal zinc finger (ZNF) domain and a C-terminal DNA binding and transposase domain. POGZ ZNF domains bind heterochromatin protein 1 (HP1) and regulate mitotic progression (Nozawa et al., 2010). Beyond POGZ’s ability to bind DNA, there is little mechanistic knowledge about POGZ’s regulation of transcription or chromatin state. It also remains unclear whether POGZ acts as a transcriptional repressor or activator. Evidence that POGZ is a transcriptional repressor is its association with HP1 proteins and the upregulation of *fetal hemoglobin* expression in *Pogz*^−/−^ hematopoetic cells (Nozawa et al., 2010; Gudmundsdottir et al., 2018). On the other hand, RNA-seq analyses of POGZ knockout brain find both down- and upregulation of gene expression (Suliman-Lavie et al., 2020); however, it is unknown whether these are due to direct or indirect effects of POGZ loss.

ASD associated variants in the DNA binding domain disrupt POGZ DNA binding activity (Matsumura et al., 2016) and reduce neurite outgrowth (Matsumura et al., 2020; Hashimoto et al., 2016; Zhao et al., 2019). POGZ homozygous deletion is embryonic lethal in mice (Gudmundsdottir et al., 2018); heterozygous and conditional knockout mice exhibit phenotypes in cortical neuron development, social and anxiety-related avoidance behaviors, and electrophysiology (Cunniff et al., 2020; Matsumura et al., 2020; Suliman-Lavie et al., 2020). Mechanisms of POGZ control of neurodevelopmental gene expression remain unexplored, and genes directly regulated by POGZ in the developing forebrain have yet to be identified.

Here, we provide a mechanistic dissection of POGZ’s function as a transcriptional regulator during embryonic forebrain development. We map POGZ bound loci in human and mouse and probe transcriptional and chromatin state phenotypes of *Pogz*^−/−^ mice. We find POGZ primarily binds euchromatic regions of the genome and acts to promote transcription and chromatin accessibility at gene REs. POGZ binds proximally (within 50kb) to genes downregulated in *Pogz*^−/−^, but not to upregulated genes, which is evidence of a direct role in gene activation and an indirect effect on gene repression. Interestingly, the top genes downregulated in *Pogz*^−/−^ are arrayed in gene clusters and encode synapse and axon guidance molecules. Our biochemical analyses reveal POGZ forms a nuclear complex with Heterochromatin Protein 1γ (HP1γ) and ADNP, another confirmed ASD risk gene. POGZ and ADNP co-occupy genomic loci and we provide evidence that relative levels of POGZ/ADNP binding at REs determine transcriptional output. Finally, to explore aspects of POGZ relevant to ASD, we analyze the transcriptional effects of heterozygous POGZ mutations in mice and identify POGZ targets in developing human cortex.

## RESULTS

### Generation of POGZ constitutive null

POGZ is expressed prenatally in the mouse cortex and ganglionic eminences (LGE and MGE; basal ganglia); *in situ* hybridization (ISH) of *Pogz* across developmental stages indicates decreasing expression in the postnatal brain (Figures S1A–S1C). In the developing cortex, *Pogz* is broadly expressed from the ventricular zone to the cortical plate, thus spanning neuronal progenitors and newborn neurons, and is more strongly expressed in neurons (Figure S1D).

**Figure 1.**
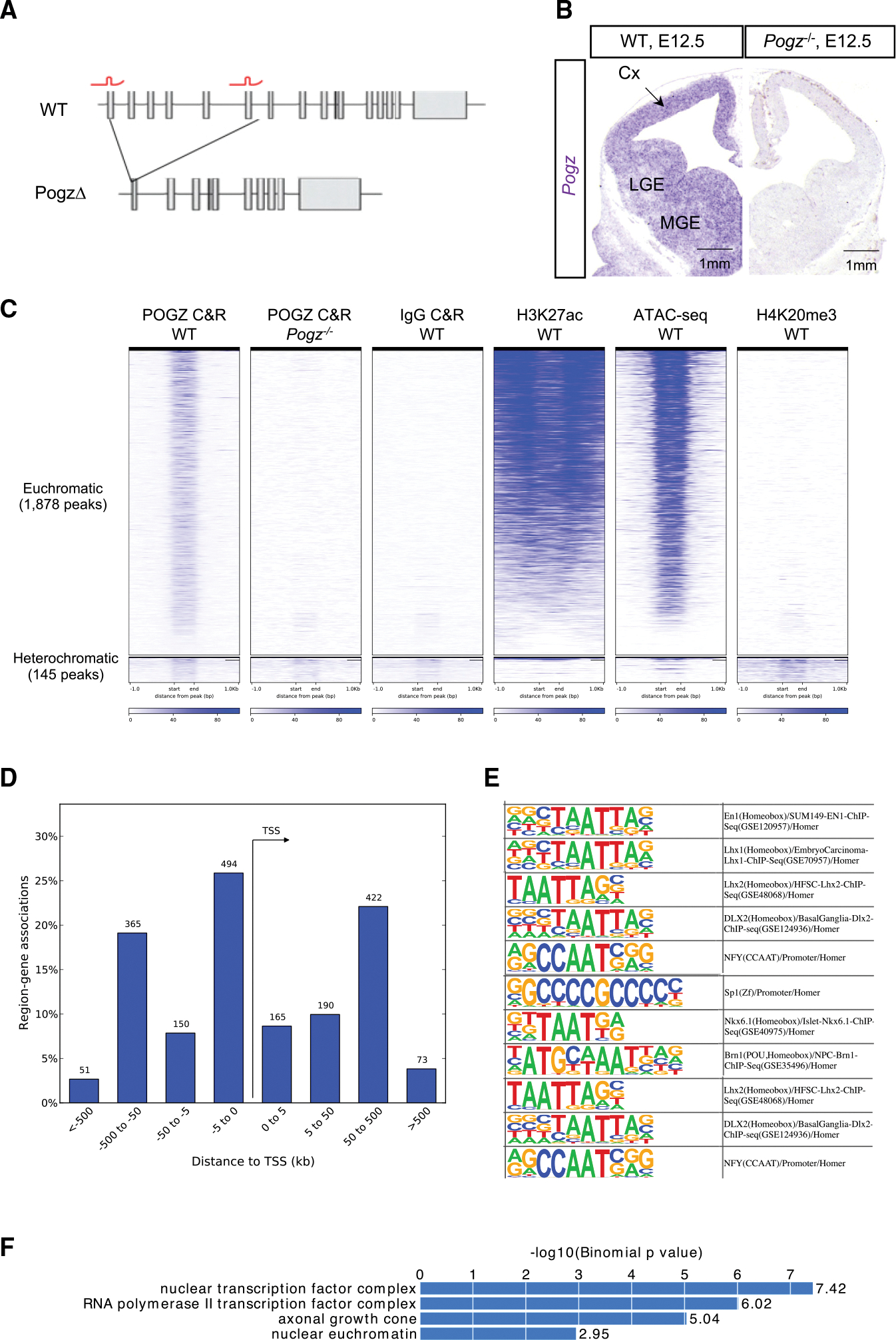
POGZ binds euchromatic loci in the developing mouse telencephalon (A) CRISPR-Cas9 generation of POGZ knockoutallele. sgRNAs (red) targeting exons 1 and 6 of mouse Pogz are indicated. Resulting 10.5 kilobase deletion of *Pogz*^−/−^ allele is shown. (B) ISH of E12.5 wild-type and *Pogz*^−/−^ telencephalon sections with *Pogz* antisense probe (purple). *Pogz* expression in MGE, LGE, and cortex is indicated. (C) Heatmap of POGZ C&R reads across consensus peaks in the mouse genome, in E13.5 telencephalon, n = 2. ATAC-seq, H3K27ac, and H4K20me3 ChIP-seq signal from E13.5 wild-type shown to the right. RPM, reads per million. (D) C&R peaks linked to nearest genes, binned by distance and orientation from gene TSS using GREAT. (E) HOMER motif analysis of POGZ C&R sequences. (F) GO terms significantly enriched in POGZ C&R nearest genes. Significant (q-value < 0.05, Benjamini-Hochberg multiple test correction) GO terms listed.

To dissect the molecular and developmental functions of POGZ, we generated a constitutive *Pogz* deletion allele. We generated founders by pronuclear injection of CRISPR-Cas9 protein and single-guide RNAs targeting exons 1 and 6, a 10 kb span (Figure 1A). Founders were screened by PCR (see STAR Methods). One founder contained a 10 kb deletion which generated a premature stop codon (Figure S1E). We outcrossed this founder to C57/Bl6 mice for ten generations. *Pogz*^−/−^ mice die at embryonic day E15.5, as previously reported, of uncertain etiology (Gudmundsdottir et al., 2018); Pogz^+/−^ mice survive and are fertile. At E13.5 we observed no *Pogz* expression in homozygous knockouts based on ISH, immunohistochemistry, and Western Blot (Figures 1B, S1D, and S1F).

We analyzed *Pogz*^−/−^ cortex at E13.5 for markers of proliferation and neurogenesis. Reduced cortical neurogenesis has been described in *Pogz* shRNA knockdown experiments in mouse embryos (Matsumura et al., 2020; Suliman-Lavie et al., 2020), and POGZ plays a role in mitosis progression in cell culture (Nozawa et al., 2010). Immunostaining for the mitotic marker phosphohistone H3 (PHH3) showed a modest increase in PHH3+ cells in the *Pogz*^−/−^ ventricular zone (Figure S2A), however the trend was not significant. We examined the ratio of cells exiting the cell cycle by labeling proliferating cells with EdU at E12.5 and collecting embryos 24 hours later; β-III Tubulin co-staining labeled the fraction of EdU positive cells that have differentiated into neurons. The fraction was decreased in *Pogz*^−/−^ however, it was not significant (p = 0.07)(Figure S2B). Furthermore, there was no significant change in the thickness of the TBR2^+^ subventricular zone or the β-III Tubulin^+^ cortical plate in *Pogz*^−/−^ (Figures S2C and S2D). No changes in cortical plate production and positioning at E13.5 were observed, nor were there changes in the approximate levels of expression of critical regulators of Layer 5 and Layer 6 fate, *Fezf2* and *Tbr1*, respectively (Figures S2E and S2F). Thus, we conclude the E13.5 *Pogz*^−/−^ cortex does not have a strong cortical neurogenesis phenotype.

### POGZ occupies euchromatin loci

As POGZ is a chromatin associated protein with a DNA binding domain, we explored its neurodevelopmental functions as a transcriptional regulator. POGZ immunostaining in mouse neuron progenitor nuclei is diffuse and euchromatic and is not restricted to DAPI-positive heterochromatin foci (Figure S3A). For an unbiased screen of POGZ occupancy genome-wide in embryonic nuclei, we performed CUT&RUN (C&R) (Skene, Henikoff, and Henikoff 2018) in E13.5 dissected wild-type mouse telencephalons using anti-POGZ antibody (100,000 nuclei per experiment, see STAR Methods). *Pogz*^−/−^ embryos and IgG were used as negative controls. We identified 2,023 consensus POGZ peaks in C&R analysis (Table S1). POGZ C&R peaks have greatly reduced signal in *Pogz*^−/−^ and wild-type IgG experiments, demonstrating the validity of POGZ interactions (Figure 1C).

To establish whether POGZ occupies euchromatic or heterochromatic loci, we compared POGZ bound loci to ATAC-seq and H3K27ac and H4K20me3 ChIP-seq we generated from wild-type E13.5 cortex. We find POGZ occupancy occurs predominantly at euchromatic rather than heterochromatic loci. Ninety-two percent of POGZ-occupied loci contain accessible chromatin and are enriched for H3K27ac, while 8% overlap peaks for the heterochromatin hallmark H4K20me3 (Figure 1C).

POGZ occupies transcription start sites (29% of peaks) and distal intergenic regions (71%), suggesting it may act as a transcriptional regulator (Figure 1D). 4.7% of POGZ C&R peaks overlap with validated enhancers that have activity in the E11.5 mouse embryonic brain (Visel et al., 2007), compared to 0.06% that overlap with non-brain enhancers, suggesting that POGZ binds brain enhancers. Furthermore, HOMER motif analysis of POGZ C&R peaks shows that they are highly enriched for homeobox (e.g., DLX, LHX, POU) and ZNF motifs (e.g., SP) of TFs that bind telencephalic enhancers (Figure 1E) (Lindtner et al., 2019; Sandberg et al., 2016). *De novo* motif discovery analysis identified putative POGZ binding motifs which will be discussed later (Figure 7).

Genes near POGZ-occupied loci are enriched for gene ontology (GO) terms that include “nuclear euchromatin” and “axonal growth cone” (Figure 1F), suggesting that POGZ may transcriptionally regulate genes that encode components of euchromatin and axon growth. Taken together, POGZ C&R and immunostaining experiments provide evidence that POGZ binds predominantly euchromatic loci and REs.

### Downregulation of neuronal and synaptic development genes in *Pogz*^−/−^ embryos

**Figure 2.**
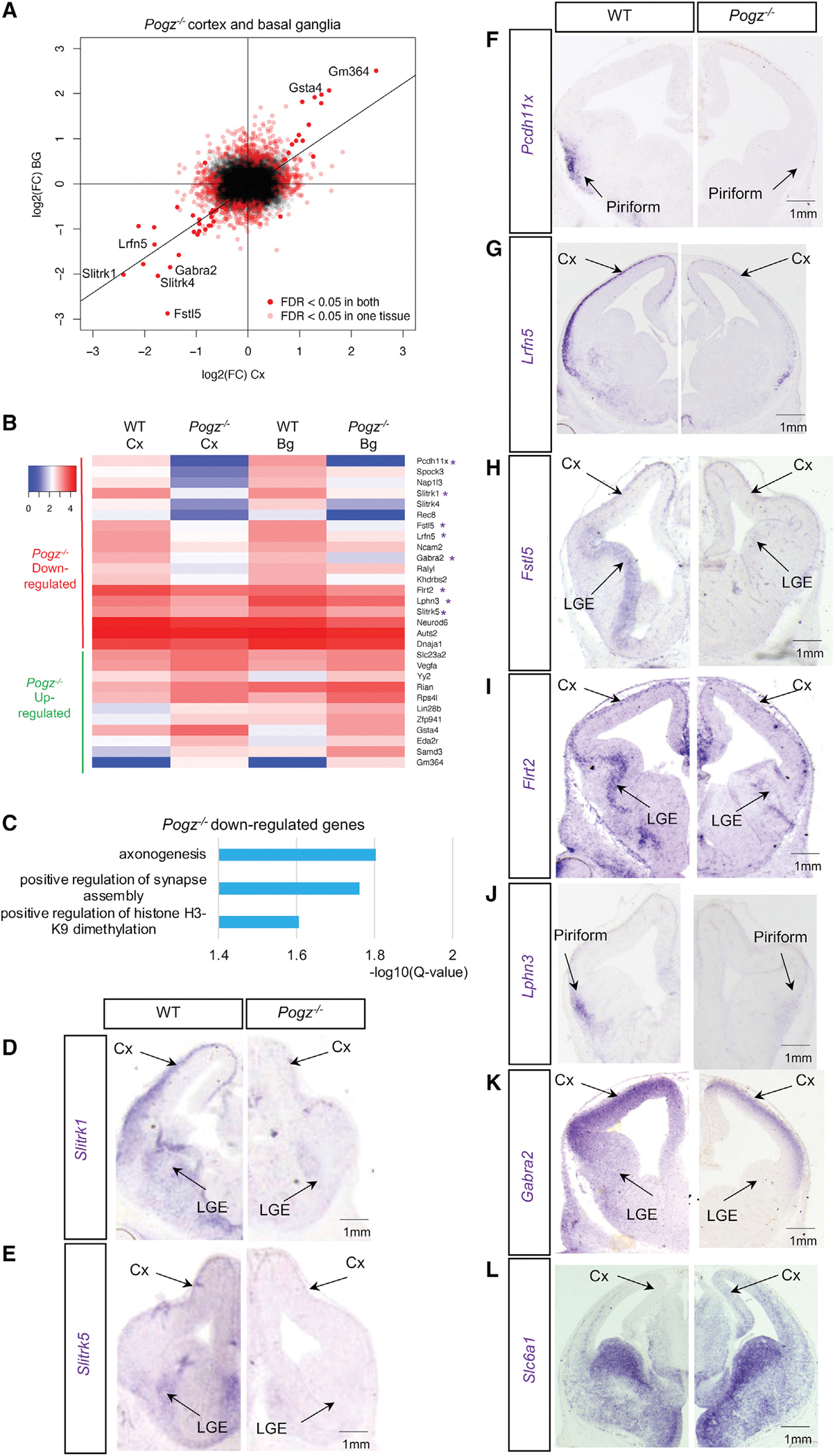
Neuronal genes downregulated in *Pogz*^−/−^ forebrain (A) Dot plot of DE genes in E13.5 *Pogz*^−/−^ cortex and basal ganglia RNA-seq compared to wild-type controls. Significant DE genes in both tissues are indicated in red dots, n = 3. Linear regression across significant DE genes in cortex and basal ganglia, adjusted R^2^ = 0.77, p = 2 × 10^−16^. (B) Heatmap of DE genes in *Pogz*^−/−^ cortex (Cx) and basal ganglia (Bg), scale is log10 of the average normalized RNA-seq reads for each gene (n = 3). Genes with a purple asterisk were validated by ISH. (C) GO analysis of *Pogz*^−/−^ downregulated genes in cortex. Significant GO terms (q-value < 0.05, Benjamini-Hochberg multiple test correction) are listed. (D–L) ISH expression and validation of genes downregulated (D-K) and upregulated (L) in *Pogz*^−/−^ at E13.5.

Toward elucidating how POGZ binding regulates gene expression, we performed RNA-seq from wild-type and *Pogz*^−/−^ littermates in E13.5 cortex and basal ganglia. We focused on these two forebrain regions because POGZ is highly expressed (Figure S1A) and they are the birthplaces of cortical excitatory and striatal inhibitory neurons, respectively, which have been implicated in ASD (Willsey et al., 2013; Parikshak et al., 2013; Satterstrom et al., 2020; Chang et al., 2015). We identified differentially expressed (DE) genes: in *Pogz*^−/−^ cortex, 177 genes were significantly downregulated and 154 were significantly upregulated (n = 3, q-value < 0.05) (Figure 2A, see Table S2). In *Pogz*^−/−^ basal ganglia, 230 genes were significantly downregulated and 267 upregulated (n = 3, q-value < 0.05). The most highly downregulated gene in both tissues was *Pcdh11x*, whose expression decreased 8-fold in cortex and 22-fold in basal ganglia. DE gene expression changes in both tissues were correlated, and there were 22 common downregulated and 11 common upregulated genes (Figures 2A and 2B).

GO analysis of downregulated genes in *Pogz*^−/−^ cortex found enrichment for the terms “axonogenesis” and “positive regulation of synapse assembly” (Figure 2C); upregulated genes were not significantly enriched for GO terms. We further examined downregulated genes linked to synapse and axonogenesis functions. Multiple *Slitrk* family genes were included in this list: in *Pogz*^−/−^ cortex, *Slitrk1, 2, 4*, and *5* were downregulated (Figure S3B), and in basal ganglia *Slitrk1, 3, 4*, and *5* were downregulated (Figure S3C). *Slitrk* genes encode leucine-rich repeat extracellular proteins that promote axon extension, excitatory synapse development, and neuronal survival (Aruga and Mikoshiba 2003; Abelson et al., 2005; Linhoff et al., 2009; Song et al., 2015; Beaubien et al., 2016). *Slitrk1* is a Tourette’s disorder candidate gene (Abelson et al., 2005; O’Roak et al., 2010) and *Slitrk5* mouse mutants have obsessive compulsive-like behaviors (Shmelkov et al., 2010). *Lrfn5*, a gene encoding another leucine-rich repeat extracellular protein implicated in NDDs (Cappuccio et al., 2019), was downregulated 3.5-fold in *Pogz*^−/−^ (Figure 2B).

*Latrophilin 3 (Lphn3)* and *Fibronectin Leucine Rich Transmembrane Protein 2 (Flrt2)* were downregulated two-fold in *Pogz*^−/−^cortex (Figure 2B). *Lphn3* encodes a GPCR that promotes excitatory synapse formation by *trans*-synaptic binding of FLRT family and Teneurin proteins (Sando, Jiang, and Südhof 2019). *Gabra2, Gabra4*, and *Gabrg1* were downregulated 2.5-fold in *Pogz*^−/−^ cortex and encode components of the GABA_A_ receptor. Genes encoding the GABA transporters SLC6A1 and SLC6A11 were upregulated in *Pogz*^−/−^ cortex; *Slc6a1* is a high confidence ASD risk gene and also associated with myoclonic atonic epilepsy/absence seizures with developmental delay (Satterstrom et al., 2020; Heyne et al., 2018). POGZ therefore regulates the transcription of genes that promote synapse formation and function, including several that are implicated in neurodevelopmental disorders.

We validated RNA-seq results by ISH in E13.5 *Pogz*^−/−^, selecting genes based on significance and fold change, as well as known involvement in synapse development and function. *Slitrk1* and *Slitrk5* were expressed in neurons of the cortex and basal ganglia and were greatly reduced in *Pogz*^−/−^ (Figures 2D, 2E, S4E, and S4F). *Pcdh11x* showed a strong reduction in gene expression in neurons of the hypothalamus, medial prefrontal cortex, and piriform cortex (Figures 2F and S4A). ISH for *Lrfn5* and *Follistatin-like 5 (Fstl5),* a gene involved in the Wnt/β-catenin pathway (Zhang et al., 2015), had reduced expression in *Pogz*^−/−^ cortex (Figures 2G, 2H, and S3F). *Lphn3* was expressed in cortical neurons and greatly reduced in *Pogz*^−/−^ (Figures 2J and S4B). *Flrt2* and *Gabra2* were expressed in neuronal layers of cortex and the lateral ganglionic eminences, where expression was reduced in *Pogz*^−/−^ (Figures 2I, 2K, S4C, and S4D). We also validated *Slc6a1* and *Cyclin D1 (Ccnd1),* transcripts whose expression increased in the cortex (Figures 2L, S3D, and S3E).

### Relationship between POGZ-regulated gene expression, POGZ binding and chromatin regulation

**Figure 3.**
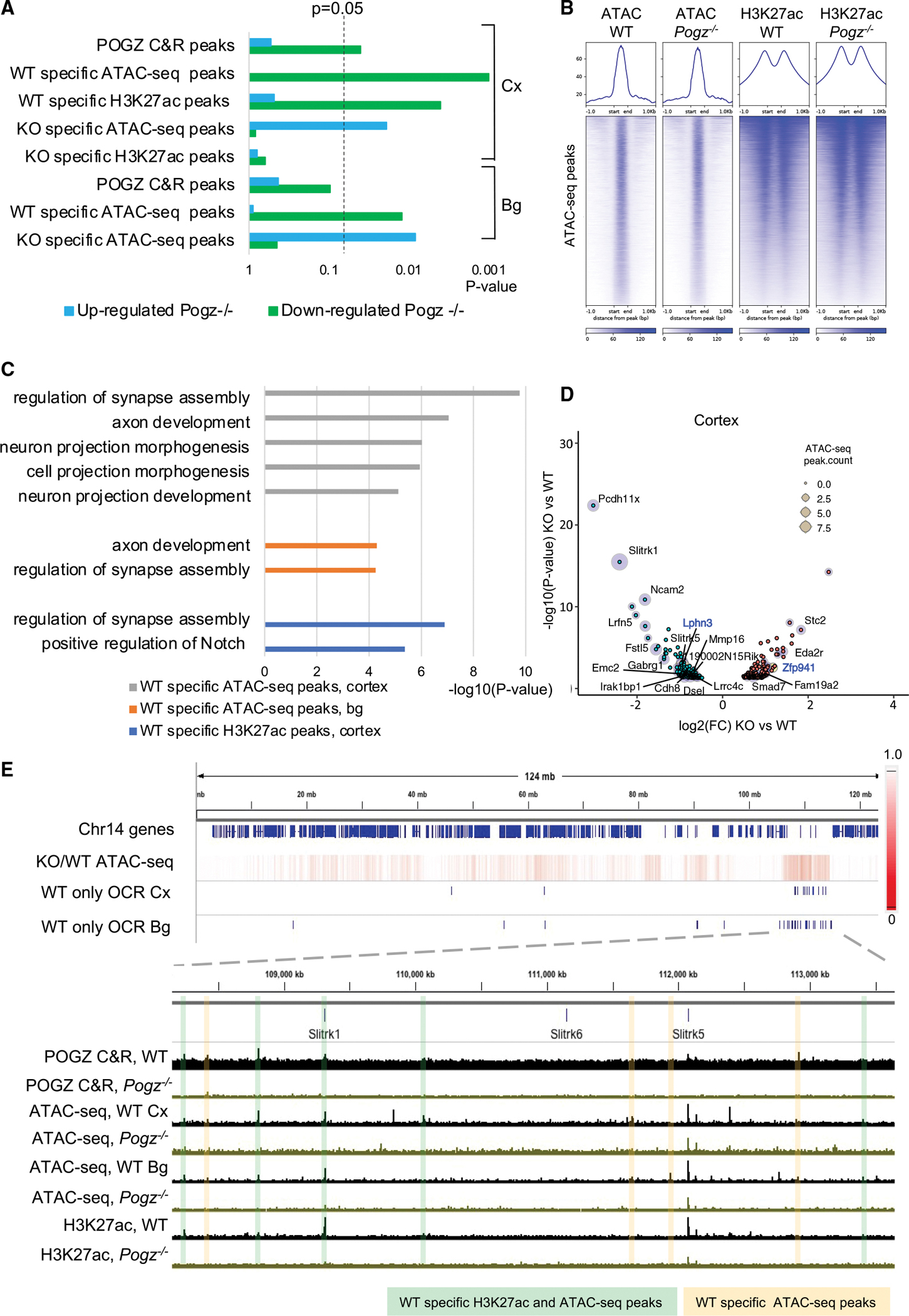
Localized changes in chromatin accessibility at DE gene loci in *Pogz*^−/−^ (A) Gene set enrichment analysis of DE genes proximal (< 50kb) to different sets of POGZ peaks: POGZ C&R peaks, and *Pogz*^−/−^ differential ATAC-seq and ChIP-seq peaks, n = 3. Up- and downregulated genes in *Pogz*^−/−^ cortex (cx) and basal ganglia (bg) were compared to all expressed genes in those tissues. (B) Plots and Heatmaps of ATAC-seq and H3K27ac ChIP-seq signal in individual wild-type and *Pogz*^−/−^ experiments at all OCRs identified in wild-type cortex at E13.5. (C) GO terms significantly enriched in genes nearest to wild-type-specific ATAC-seq peaks in E13.5 cortex and basal ganglia (bg), and wild-type-specific H3K27ac ChIP-seq peaks in cortex. Significant GO terms (q-value < 0.05, Benjamini-Hochberg multiple test correction) are listed. (D) Volcano plot of DE genes at E13.5 *Pogz*^−/−^ cortex. Upregulated (red dot) or downregulated (green dot) genes that contain proximal ATAC-seq peaks that are *Pogz*^−/−^ or wild-type specific, respectively, are indicated with blue halos. (E) Genome browser view of all genes on mouse chromosome 14. Heatmap of ATAC-seq reads in *Pogz*^−/−^ cortex normalized to wild-type in 50 kb genomic bins. Blue hashes are wild-type-specific OCRs in cortex (Cx) and basal ganglia (Bg). Highlighted POGZ occupied peaks at the *Slitrk1* and *Slitrk5* locus are wild-type specific OCRs (yellow) or are both wild-type specific OCRs and wild-type specific H3K27ac peaks (green). Tracks are sequencing reads from individual C&R, ATAC-seq and ChIP-seq experiments at E13.5.

Our RNA-seq analyses suggested that POGZ acts as both an activator and a repressor. To explore the relationship between POGZ binding and DE genes in *Pogz*^−/−^, we performed gene set enrichment analysis for genes proximal to POGZ C&R peaks. Downregulated genes in *Pogz*^−/−^ cortex are significantly enriched within 50kB of POGZ C&R peaks (odds ratio = 1.49, p = 0.04, Fisher’s exact test), while upregulated genes are not significantly enriched (odds ratio = 1.05, p = 0.53, Fisher’s exact test) (Figure 3A); all expressed genes in wild-type cortex were used as controls (see Methods). This provides evidence that POGZ binding is associated with the transcription of genes downregulated in *Pogz*^−/−^.

To investigate whether POGZ regulates chromatin to modify gene expression, we performed assay for transposase-accessible chromatin (ATAC-seq) in *Pogz*^−/−^ to map changes in chromatin accessibility genome-wide. In addition, we performed H3K27ac ChIP-seq in *Pogz*^−/−^ to assess whether POGZ regulates the deposition of this mark of active enhancers (Rada-Iglesias et al., 2011). We observed similar ATAC-seq and H3K27ac levels in wild-type and *Pogz*^−/−^ (Figure 3B) and conclude that there are no overt genome-wide differences in chromatin accessibility or H3K27ac deposition.

Instead, we found that changes in chromatin state were restricted to a small subset of genes and REs. Gene body chromatin accessibility levels were reduced over downregulated genes compared to unchanged genes (p = 2.2×10^−16^, Student’s t test)(Figure S5A). We performed differential peak calling to identify open chromatin regions (OCRs) that are specific to wild-type or *Pogz*^−/−^ . In cortex, 277 wild-type specific OCRs and 1,835 *Pogz*^−/−^-specific OCRs were identified (n = 3, Table S3); in basal ganglia 405 wild-type-specific OCRs and 206 *Pogz*^−/−^-specific OCRs were identified (n=3). Differential OCRs in cortex and basal ganglia were highly overlapping: 22% of wild-type-specific cortex OCRs overlap with basal ganglia, and 4% of *Pogz*^−/−^-specific cortex OCRs overlap with basal ganglia (permutation analysis, p = 0.001) (Figures S5B and S5C).

GO analysis on genes nearest wild-type specific OCRs yielded terms such as “regulation of synapse assembly” and “axon development” (Figure 3C), which matches *Pogz*^−/−^ downregulated gene terms (Figure 2C). No significant GO terms were found for genes nearest *Pogz*^−/−^-specific OCRs. We asked whether wild-type and *Pogz*^−/−^-specific OCRs are linked to DE genes. Gene set enrichment analyses found *Pogz*^−/−^ downregulated genes are significantly enriched within 50kb of wild-type specific OCRs (odds ratio = 5.59, p = 0.001 for cortex; odds ratio = 2.73, p = 0.012 for basal ganglia, Fisher’s exact test). Upregulated genes are also enriched proximal to *Pogz*^−/−^-specific OCRs (odds ratio = 2.54, p = 0.019 for cortex; odds ratio = 2.56, p = 0.008 for basal ganglia, Fisher’s exact test)(Figure 3A). All the most significantly downregulated genes are located proximally to wild-type-specific OCRs, while a subset of significantly upregulated genes are located proximally to *Pogz*^−/−^-specific OCRs (Figure 3D). These analyses show that there is an association between changes in gene expression in *Pogz*^−/−^ with changes in chromatin accessibility at proximal OCRs.

Next, we asked whether changes in H3K27ac deposition are associated with *Pogz*^−/−^ DE genes. Differential peak calling identified 1,973 wild-type specific and 521 *Pogz*^−/−^-specific H3K27ac ChIP-seq peaks in cortex (n = 2, Table S3). GO analysis of wild-type specific H3K27ac peaks found terms such as “positive regulation of synapse assembly” enriched (Figure 3C). Gene set enrichment analysis found that downregulated genes are significantly enriched proximal to wild-type specific H3K27ac peaks (odds ratio = 1.90, p = 0.004, Fisher’s exact test), while upregulated genes are not significantly enriched proximal to *Pogz*^−/−^-specific H3K27ac peaks (Figure 3A). Thus, our integration of RNA-seq, ChIP-seq, and ATAC-seq analyses concluded that changes in chromatin state in *Pogz*^−/−^ are localized near DE genes.

We examined individual examples of chromatin state changes at DE gene loci in *Pogz*^−/−^. The *Slitrk1* and *Slitrk5* genes and surrounding gene desert (Figure 3E) contain the greatest decreases in chromatin accessibility on chromosome 14. Similarly, the greatest decreases in chromatin accessibility on chromosome 5 are at the *Gabra2*, *Gabrg1*, and *Gabra4* genes, as well as the *Lphn3* gene locus (Figure S5D). Other examples include *Pcdh11x* and *Nap1l3* on the X chromosome (Figure S5E). Notably, seven of the top fifteen downregulated genes are found in gene clusters (within 100kb) with other downregulated genes: *Pcdh11x* and *Nap1l3*, *Slitrk1* and *Slitrk5*, *Gabra2*, *Gabrg1*, and *Gabra4*.

We tested POGZ C&R peaks that were wild-type specific OCRs and H3K27ac^+^ for enhancer activity. Two elements at the *Slitrk1* and *Slitrk5* locus had enhancer activity in a luciferase transcription assay in primary cultures from embryonic cortex and basal ganglia (Figures S6A and S6B). Two POGZ bound loci proximal to the *Lphn3* locus had enhancer activity as well (Figures S6C and S6D). Co-transfection of POGZ did not affect candidate RE enhancer activity (Figure S6C), suggesting that POGZ activity at these REs may depend on the chromatin environment of the developing mouse cortex and basal ganglia. These experiments provide evidence that elements whose chromatin accessibility and H3K27ac levels are positively regulated by POGZ are transcriptional enhancers.

### POGZ and ADNP form a complex with HP1γ and co-occupy loci

**Figure 4.**
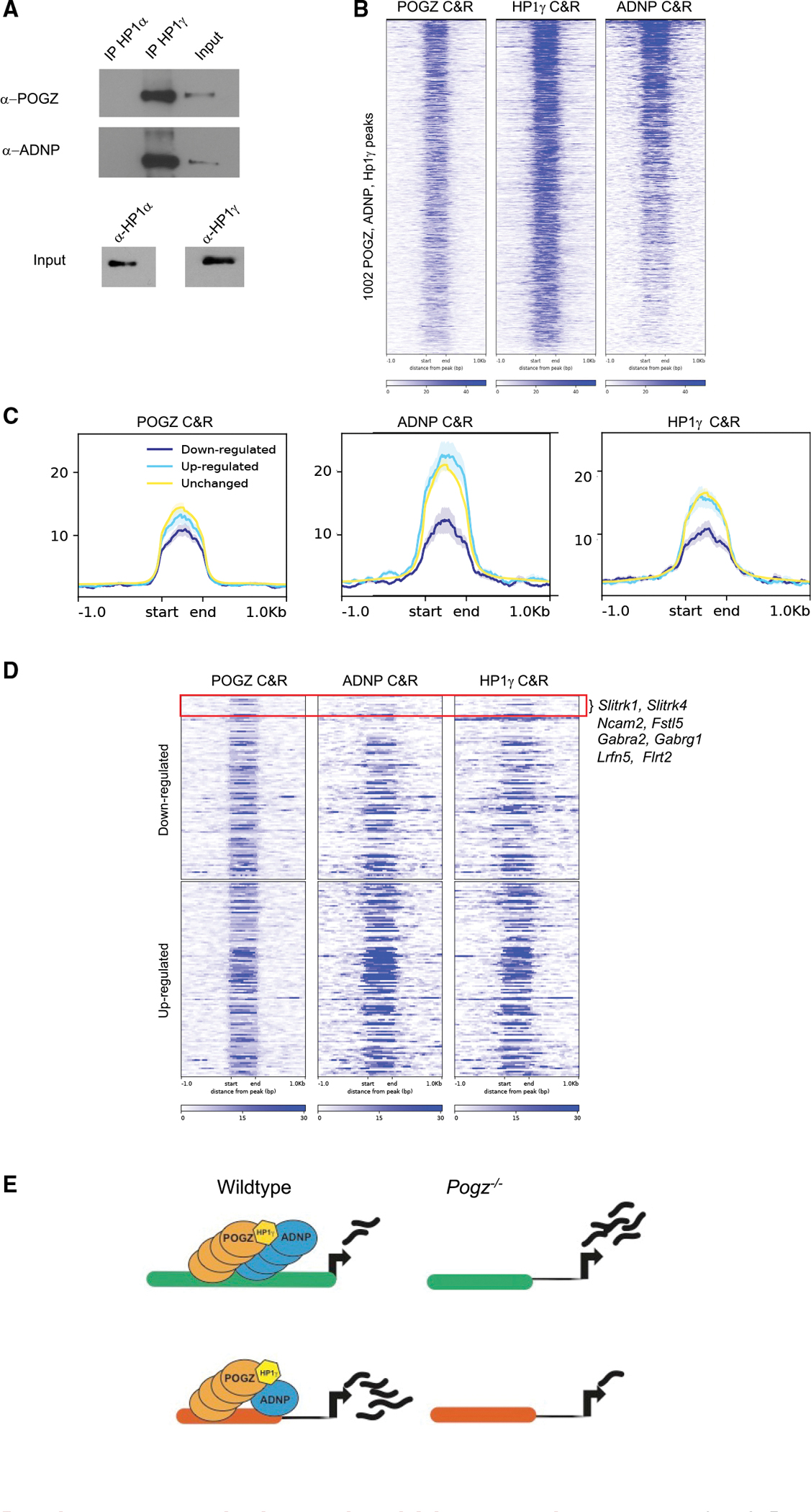
POGZ and ADNP form a nuclear complex with HP1γ (A) Western blots for POGZ and ADNP in IPs for HP1α and HP1γ from nuclear extracts of E13.5 wild-type mouse cortex (B) Heatmap of POGZ, ADNP, and HP1γ C&R reads across consensus peaks for all three antibodies in E13.5 mouse forebrain, n = 2. (C) POGZ,ADNP, and HP1γ C&R signal overPOGZ C&R peaks in three different segments of the genome: proximal(within 100kb) to downregulated genes (q-value < 0.01), proximal to upregulated genes (q-value < 0.01), proximal to unchanged genes. (D) Heatmap of POGZ, ADNP, and HP1γ C&R reads across consensus peaks proximal (within 100 kb) to *Pogz*^−/−^ DE genes. (E) Proposed model for POGZ, ADNP, and HP1γ activity at REs. High levels of binding of all three proteins leads to repression of proximal genes (observed upregulation in *Pogz*^−/−^), while high levels of POGZ and reduced levels of ADNP and HP1γ leads to activation of proximal genes (and observed downregulation in *Pogz*^−/−^).

Our observations that POGZ binds euchromatin and promotes chromatin accessibility and gene expression suggests that its dominant functional interaction may not be with repressive heterochromatin. Previously POGZ has been shown to interact with heterochromatin protein 1 (HP1), a structural component of heterochromatin (Nozawa et al., 2010). HP1 variants α and β localize primarily in heterochromatin, while γ is found in both heterochromatin and euchromatin and at actively transcribed genes (Canzio, Larson, and Narlikar 2014; Vakoc et al., 2005; Minc, Courvalin, and Buendia 2000). We asked which HP1 variant POGZ binds in nuclear extracts of E13.5 mouse cortex. By co-immunoprecipitation (co-IP) we found HP1γ antibody pulled down POGZ, whereas HP1α did not (Figure 4A).

HP1γ interacts with ADNP and CHD4 to form the ChAHP complex that represses gene transcription by generating inaccessible chromatin (Ostapcuk et al., 2018). ADNP is a high-confidence ASD risk gene (Satterstrom et al., 2020) that recognizes DNA motifs through its homeodomain and directs binding of the ChAHP complextoeuchromatin.WetestedwhetherHP1variantsinteract with ADNP in E13.5 cortex. As we saw for POGZ, HP1γ co-IP’d with POGZ, whereas HP1α did not (Figure 4A).

Next, we assessed whether POGZ, HP1γ, and ADNP occupy the same chromosomal loci in E13.5 mouse telencephalon using the C&R assay with anti-HP1γ and anti-ADNP antibodies. Computational analysis found 1,002 consensus loci occupied by all three proteins (permutation analysis, p = 0.001)(Figures 4B and S6E). ATAC-seq and ChIP-seq from E13.5 cortex showed these loci are euchromatic, with accessible chromatin, enrichment for H3K27ac, and had no detectable H3K9me3 heterochromatin (Figure S6F). These loci were located at transcription start sites and enriched for GO terms such as “pallium development,” “gliogenesis,” and “ephrin receptor signaling,” which suggests that shared targets are proximal REs of genes that regulate cortical development (Figure S6G).

As ADNP is a repressor of neuronal gene expression (Ostapcuk et al., 2018), we wondered whether ADNP is present where POGZ promotes neuronal gene expression. Interestingly, we found reduced co-occupancy of ADNP and HP1γ with POGZ at loci proximal to genes downregulated in *Pogz*^−/−^, compared to genes that were upregulated or unchanged (Figure 4C). Examples of POGZ occupied loci where ADNP and HP1γ binding was reduced are the *Slitrk* family and GABA_A_ receptor genes (Figure 4D). We propose that POGZ acts as a positive regulator of transcription when it occupies loci with reduced co-occupancy of ADNP and HP1γ, and acts as a repressor with high co-occupancy of ADNP and HP1γ (Figure 4E).

### *Pogz* heterozygote mice have reduced *Adnp* expression

Our genetic analyses of *Pogz*^−/−^ mice revealed a crucial role for POGZ in promoting the transcription of specific neurodevelopmental genes. Our biochemical analyses revealed that POGZ directly binds REs proximal to these gene loci and that it forms a nuclear complex which co-occupies genomic loci together with HP1γ and ADNP. However, ASD is caused by heterozygous loss-of-function POGZ variants. Thus, we searched for phenotypes in *Pogz*^+/−^ mice that express half the amount of POGZ protein (Figures S7A and S7B). To look for gross anatomical defects, we performed MRI on P28 *Pogz*^+/−^ brains and found no significant change in cortex size (Figure S7C).

**Figure 5.**
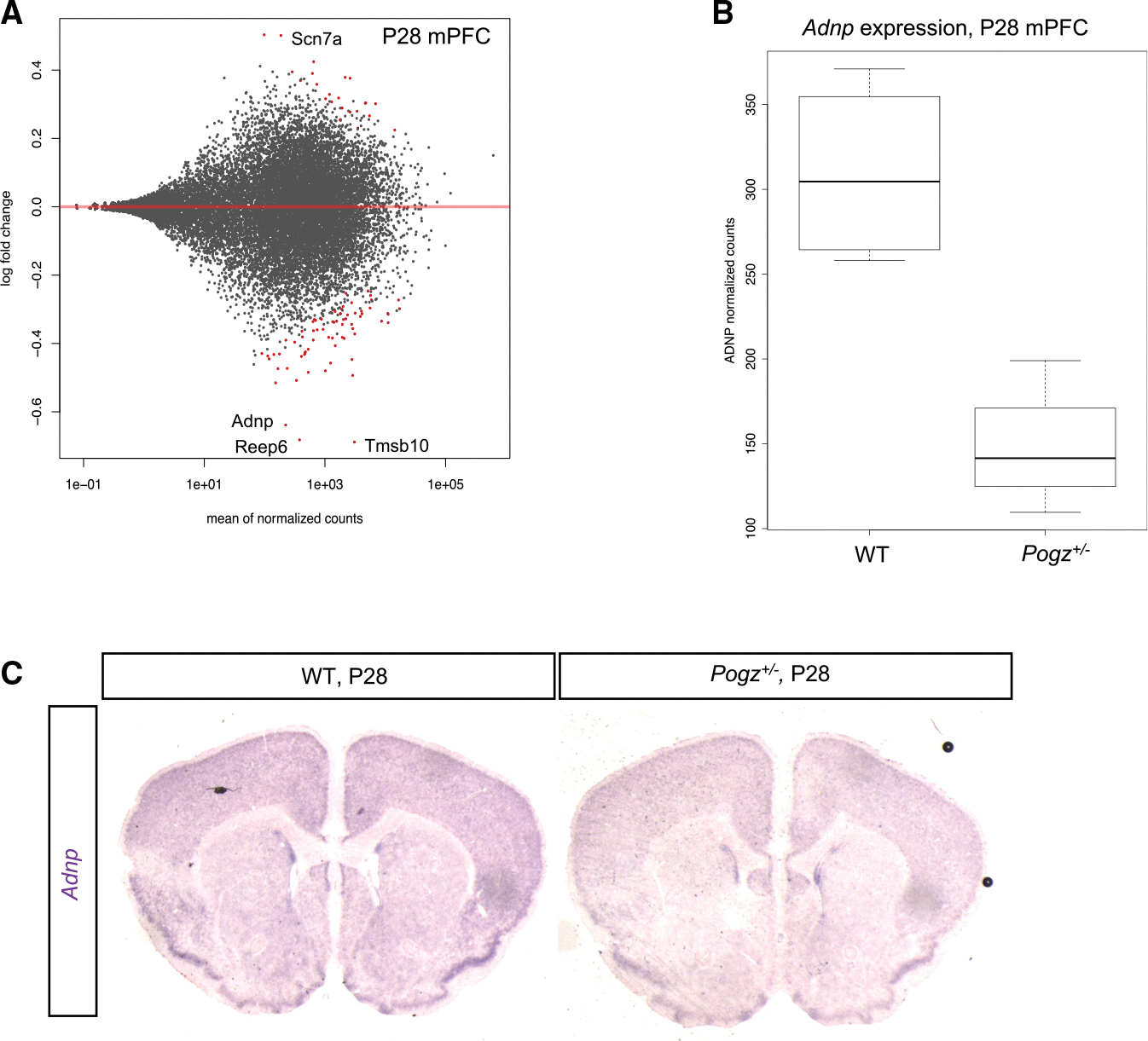
*Adnp* expression reduced in *Pogz* heterozygote (A) MA plot showing differentially expressed genesin P28 *Pogz*^+/−^ mPFC RNA-seq counts compared to wild-type. Significant DE genes (q-value < 0.05) are indicated in red dots. (B) Normalized *Adnp* RNA-seq read counts in wild-type and *Pogz*^+/−^ mPFC. (C) ISH using antisense *Adnp* RNA probe in wild-type and *Pogz*^+/−^ P28 coronal sections.

Pogz^+/−^ mice have electrophysiological phenotypes in mPFC (Cunniff et al., 2020); thus, we analyzed mPFC of P28 *Pogz*^+/−^. We performed RNA-seq in *Pogz*^+/−^ and wild-type mice, and found 29 significantly downregulated and ten upregulated genes (q-value < 0.05, n = 4)(Table S4). Interestingly, *Adnp* was one of the most downregulated genes (1.5-fold, q-value = 0.0008)(Figures 5A and 5B). ISH confirmed reduction of *Adnp* mRNA levels in *Pogz*^+/−^ cortex at P28 (n = 3)(Figure 5C).

Next, we investigated P0 and P10 Pogz^+/−^ cortex for transcriptomic changes, and similarly to P28, we observed only few changes in gene expression. At P0, 16 genes were downregulated and 14 upregu lated (q-value < 0.05, n = 3), and at P10 one gene was downregulated and 3 upregulated (q-value < 0.05, n = 3)(Figures S7D and S7E; Table S4). At these ages, a change in *Adnp* mRNA was not detected by RNA-seq. The subtle and variable transcriptomic effect of heterozygosity is unsurprising considering that in the homozygote null at E13.5, we likewise observed few strong changes in gene expression and/or chromatin accessibility; although, when present, changes in gene expression and chromatin accessibility were highly correlated.

### POGZ-occupied loci in human fetal cortex are euchromatic and enriched for NDD genes

**Figure 6. ):**
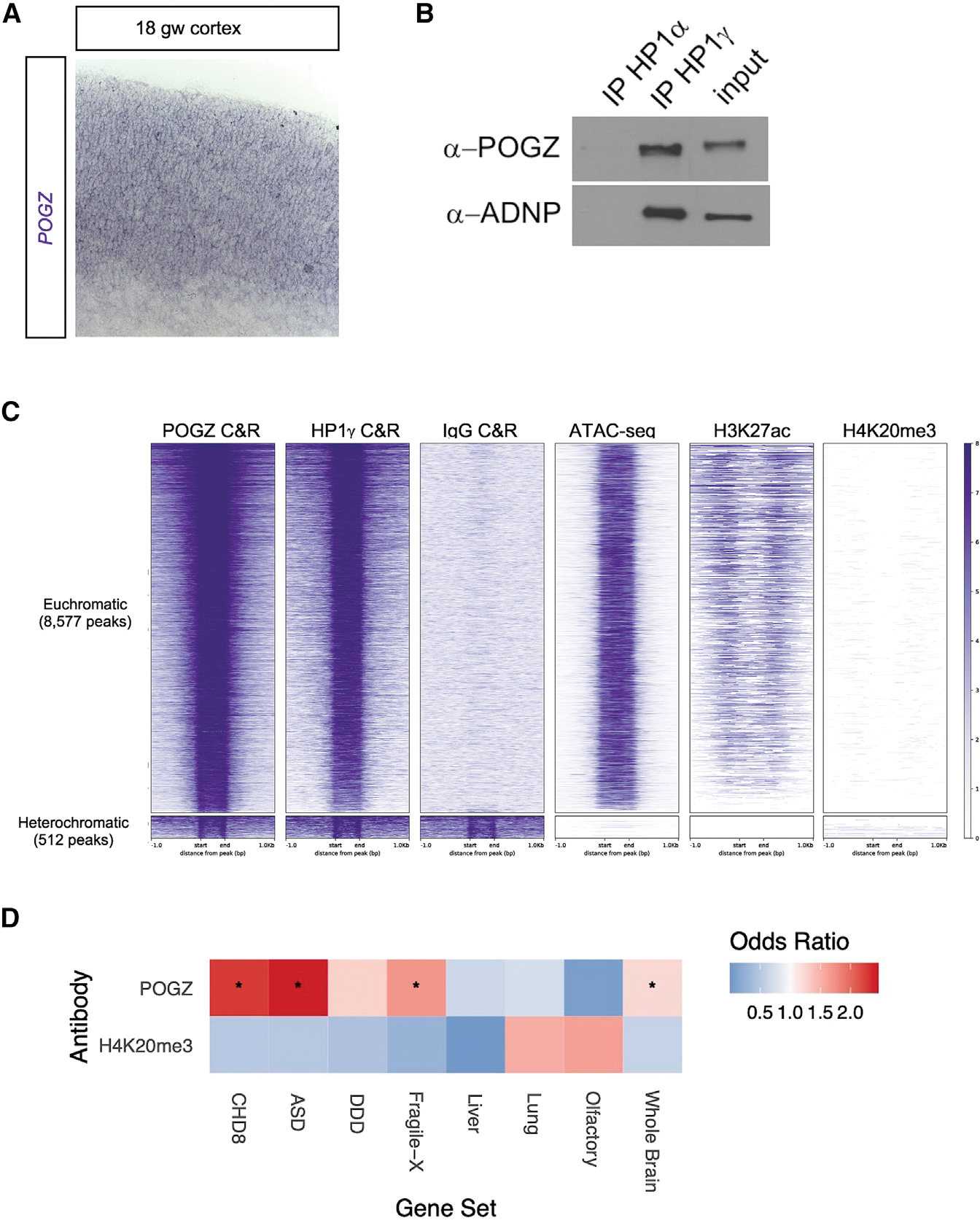
POGZ binds euchromatic loci and HP1γ in human fetal cortex (A) ISH of *POGZ* antisense probe (purple) in 18 gw fetal cortex tissue (B) Co-IPs for HP1α and HP1γ from nuclear extracts of 18 gw cortex. (C) Heatmap of POGZ, HP1g, and IgG control C&R reads across POGZ consensus peaks (n = 9,089) in human mid-fetal cortex. ATAC-seq and H3K27ac and H4K20me3 ChIP-seq reads from mid-fetal cortex samples (Markenscoff-Papadimitriou et al., 2020). RPM reads per million. (D) Gene set enrichment analysis for genes proximal (within 50 kb) to POGZ C&R peaks, H4K20me3 peaks as control. Gene sets that are significantly enriched in POGZ C&R-proximal genes are indicated with a star (q-value < 0.001 after multiple testing correction).

To investigate whether our findings in mice are applicable to human, we analyzed POGZ occupancy in human fetal cortex. *POGZ* mRNA is expressed in the mid-fetal human cortex (Figure 6A). As in the mouse, HP1γ antibody pulled down POGZ in nuclear extracts from 17 gestation week (gw) cortex (Figure 6B). We studied POGZ occupancy by CUT&RUN in nuclei isolated from 17 gw and 18 gw human cortex and identified 9,089 consensus peaks (n = 3, 10 million nuclei). POGZ-occupied loci exhibited high levels of chromatin accessibility in ATAC-seq data from human fetal cortex (Markenscoff-Papadimitriou et al., 2020). Eighty-nine percent of POGZ C&R peaks overlap with an ATAC-seq peak, compared to 3.5% that overlap shuffled peaks. As in the mouse, C&R for HP1γ and ChIP-seq for histone modifications show POGZ and HP1γ primarily overlapped H3K27ac peaks and not H4K20me3 heterochromatin modifications (Figure 6C). Paralleling findings in mouse cortex, POGZ was localized at distal loci in the *SLITRK1* and *SLITRK5* cluster (Figure S7F). These experiments provide evidence that POGZ co-occupies euchromatic loci with HP1γ in both human and mouse.

Next, we asked whether POGZ occupies gene loci implicated in NDDs. We performed gene set enrichment analysis for genes proximal (within 50 kb) to POGZ C&R peaks in human mid-fetal cortex. We tested various NDD relevant gene sets: ASD and Developmental Delay risk genes (Satterstrom et al., 2020; Deciphering Developmental Disorders Study 2017), and targets of the ASD gene CHD8 and Fragile-X Syndrome gene FMRP (Cotney et al., 2015; Darnell et al., 2011), and compared enrichment to control gene sets. POGZ C&R peak-proximal genes were significantly enriched for ASD risk genes and targets of CHD8 and FMRP (q-value < 0.001, Benjamini-Hochberg multiple test correction), and the enrichment was greater than a control set of all expressed protein-coding genes (Figure 6D). Furthermore, the proportion of CHD8 target genes proximal to POGZ C&R peaks was statistically significant compared to the proportion of 1,000 random samples of whole brain expressed genes (empirical p value < 0.001). From these analyses we concluded that POGZ occupied loci in the mid-fetal human cortex are significantly enriched for ASD risk genes and CHD8 target genes.

### POGZ occupied loci are enriched for Transposable Elements (TEs)

**Figure 7.**
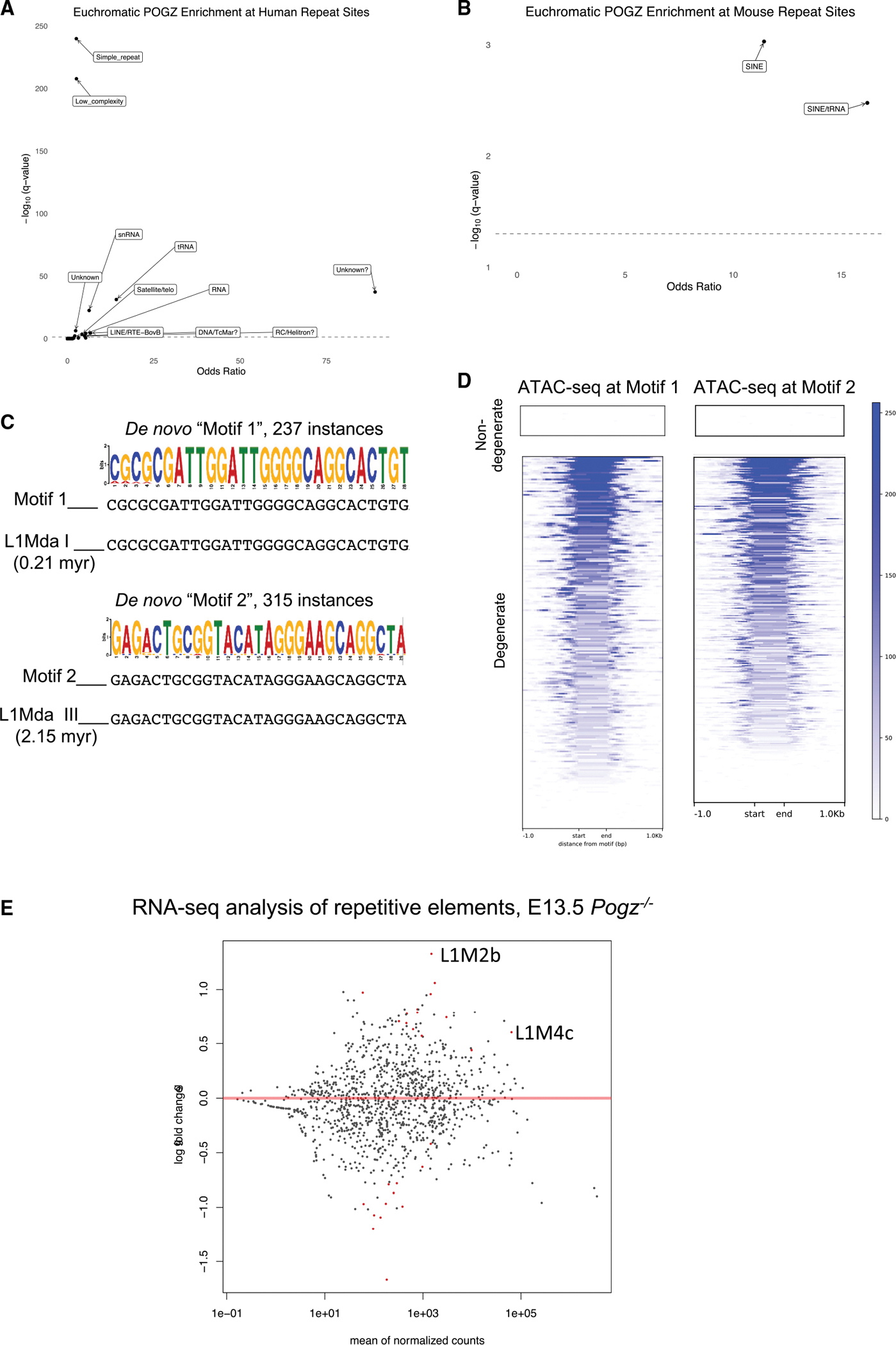
POGZ occupied loci enriched for TEs, L1 retrotransposon motifs (A and B) Volcano plots of TEs enriched in euchromatic POGZ C&R peaks from human 18 gw cortical (A) and mouse E13.5 telencephalon (B) samples. Elements above the dotted line (q-value < 0.05 after multiple testing correction) are significantly enriched. (C) Repeatmasker alignment of two *de novo* POGZ binding motifs identified by MEME-CHIP analysis of POGZ C&R data. (D) Plot and Heatmap of ATAC-seq signal at instances of predicted POGZ DNA-binding motifs 1 and 2 in POGZ C&R peaks. POGZ peaks are clustered by whether there is no mismatch with the motif sequence (non-degenerate) or whether there are mismatches (degenerate). (E) MA plot showing differentially expressed repetitive elements in *Pogz*^−/−^ E13.5 RNA-seq data, significant DE genes (q-value < 0.05) are indicated in red dots.

Because POGZ contains a *pogo* transposon sequence in the final exon, we wondered whether POGZ-occupied loci are enriched for TEs. We asked if any classes of TEs are enriched in euchromatic POGZ C&R loci from the developing mouse telencephalon and human cortex. In human, LINE elements were enriched; in mouse, SINE elements and tRNA-derived SINEs were enriched (q-value < 0.05 after multiple testing correction)(Figures 7A and 7B).

*De novo* motif analysis of mouse POGZ C&R peaks identified one 28bp and one 29bp motif that mapped to the promoters of L1Mda-1 and L1Mda-III, two of the most recently evolved L1 LINE genes in the mouse genome (Figure 7C) (Sookdeo et al., 2013). Nondegenerate instances of these motifs in the C&R peaks are heterochromatic, while degenerate instances of these motifs are euchromatic and have accessible chromatin, suggesting they are at REs (Figure 7D).

Given the enrichment of L1 motifs in POGZ bound sites, we wondered whether POGZ regulates L1 transcription. We revisited *Pogz*^−/−^ RNA-seq data from E13.5 cortex and quantified differences in transcription of repetitive elements. Eight genes had significantly increased expression (q-value < 0.05) in *Pogz*^−/−^, including two L1 genes; the most upregulated gene (2.5 fold) was the retrotransposon L1M2b (Figure 7E). Taken together, our analyses conclude that POGZ C&R loci are enriched for TEs and L1 sequence motifs, and that POGZ modulates L1 transcription in developing mouse cortex.

## DISCUSSION

Pathogenic variants in POGZ are strongly associated with risk for NDDs such as ASD, ID, and Developmental Delay (Satterstrom et al., 2020; Deciphering Developmental Disorders Study, 2015). Here, we generated a constitutive *Pogz* null allele to probe the molecular functions of POGZ in mouse neuronal development. We performed chromatin and gene expression profiling in *Pogz*^−/−^ embryos and mapped POGZ occupancy genome-wide in the embryonic mouse telencephalon and fetal human cortex. Our analyses conclude that POGZ binding to promoters and distal enhancers promotes chromatin accessibility and transcription at a highly specific set of genes, many of which encode synaptic proteins and axon guidance molecules. In addition, there are genes which are upregulated in *Pogz*^−/−^ and have increased chromatin accessibility at proximal REs, though POGZ binding is not enriched at these loci. We provide evidence that POGZ is in a repressive nuclear complex with HP1γ and ADNP, the latter being a high-confidence ASD risk gene. Below, we discuss the implications of our findings on POGZ function and speculate on an evolutionary role of POGZ.

### POGZ regulates euchromatin

One of the main conclusions of our analysis is that POGZ occupies euchromatic as well as heterochromatic loci and, depending on its binding context, acts to either promote or repress transcription. Genome-wide mapping of POGZ occupancy by C&R in developing mouse (Figure 1) and human (Figure 6) brain tissues revealed it predominantly binds euchromatic loci. POGZ C&R peaks are found at transcription start sites and distal REs containing accessible chromatin and the active RE mark H3K27ac. We validated the enhancer function of two distal REs bound by POGZ using luciferase assays; furthermore, 96 validated enhancers with activity in embryonic brain tissue (Visel et al., 2007) overlap POGZ C&R peaks. Thus, like the ASD risk genes CHD8 and ADNP (Cotney et al., 2015; Ostapcuk et al., 2018), POGZ binds REs.

POGZ is often cited as a transcriptional repressor following evidence demonstrating POGZ interaction with HP1, a chromatin scaffolding protein associated with repressive heterochromatin (Nozawa et al., 2010). While this work showed POGZ binds multiple subtypes of HP1, we found POGZ preferentially binds HP1γ in nuclear extracts from developing mouse and human cortex (Figure 4, Figure 6). This discrepancy may be attributed to our co-IP experiments being performed in developing cortical tissues, whereas the previous study was performed in 293 and HeLa cells. Thus, it is possible that POGZ has a differential affinity for HP1 subtypes in different cellular contexts. Interestingly, numerous missense ASD-associated variants have been identified in the HP1-binding ZNF domain of POGZ (Sanders et al., 2015; Satterstrom et al., 2020; Stessman et al., 2016) These POGZ variants may point to the interaction with HP1γ being relevant to the pathology of ASD.

### POGZ promotes neuronal gene transcription and chromatin accessibility at gene clusters

In accordance with its role as an ASD gene, we found that POGZ promotes the expression and chromatin accessibility of a subset of genes known to play a role in synapse formation and function. Interestingly, both in the cortex and the basal ganglia, the set of genes showing the most differential expression in *Pogz*^−/−^ were identical (Figure 2). This suggests that POGZ has similar functions in cortex and basal ganglia. However, over developmental time (E13, P0, P10, P28), observed transcriptomic changes evolved, suggesting that POGZ’s targets shift at different developmental stages. Alternatively, because the transcriptomic changes in the E13.5 homozygotes are much more apparent than in the P0, P10, and P28 heterozygotes, the consistency of gene expression changes may be obscured.

Mechanistic insight into how POGZ functions to promote gene expression was gained by ATAC-seq and ChIP-seq analysis of *Pogz*^−/−^ cortex and basal ganglia. We found the effects of POGZ deletion on chromatin accessibility and H3K27ac deposition were highly restricted to the *Pogz*^−/−^ DE gene loci (Figure 3). This highly localized effect of mutation of an NDD-linked transcriptional regulator is unusual: *Chd8, Mecp2*, and *Tbr1* mutants have more diffuse effects on gene expression (Gompers et al., 2017; Chahrour et al., 2008; Fazel Darbandi et al., 2018).

A striking feature of genes regulated by POGZ is that 50% of the most highly downregulated genes are located in proximity to each other in small clusters: *Slitrk1* and *Slitrk5; Pcdh11x* and *Nap1l3; Gabra2, Gabrg1,* and *Gabra4*. This evidence, coupled with the observation that chromatin accessibility losses are restricted to these loci, is reminiscent of β-globin regulation in erythroid cells. There, the locus control region (LCR) coordinates multiple β-globin genes by recruiting chromatin-modifying, co-activator, and transcription factor complexes (Fang et al., 2003). Intriguingly, POGZ is required for fetal hemoglobin expression, although direct regulation of the β-globin locus or LCR by POGZ has not been shown (Gudmundsdottir et al., 2018). We speculate that POGZ may regulate the clustered neuronal genes in a similar manner. REs that are regulated by POGZ in these gene clusters may be LCRs coordinating mutually exclusive gene expression patterns. Some of the candidate REs regulated by POGZ can drive transcription in cell culture (Figure S6), and thus their *in vivo* functions merit further study.

We did not detect a cortical neurogenesis phenotype at E13.5, which was surprising given other studies that found POGZ knockdown affecting embryonic cortical neurogenesis and adult neurogenesis in the dentate gyrus (Matsumura et al., 2020; Suliman-Lavie et al., 2020). We can attribute this discrepancy to differences in the methods of POGZ knockdown used (in utero electroporation of short hairpin RNAs versus our analysis of constitutive POGZ knockout mice), and the ages analyzed (E16.5 and adult versus our E13.5 analysis). Mitotic index and cortical plate thickness were normal in E13.5 *Pogz* homozygotes, and cortical surface area and volume were normal in P28 *Pogz* heterozygotes, suggesting that there was not a large effect on proliferation and neurogenesis (Figure S2). Thus, the effects on neuronal gene expression that we observe appear to be due to POGZ function in postmitotic neurons rather than defects in cortical neurogenesis.

### Convergent genomic targets of ASD risk genes POGZ, ADNP, and CHD8

Our C&R experiments showed that half of POGZ occupied loci are co-occupied by ChAHP complex members HP1γ and ADNP (Figure 4). ADNP is a high-confidence ASD risk gene and a repressor of neuronal lineage gene expression; *ADNP*^−/−^ ES cells and mouse embryos have defects in neuronal fate specification (Ostapcuk et al., 2018; Pinhasov et al., 2003). Because ChAHP binding is markedly reduced at loci where POGZ promotes gene expression (Figure 4), we propose that at loci co-occupied by POGZ and ADNP/ChAHP, ADNP antagonizes POGZ’s activating potential. The 50% reduction of *Adnp* RNA in *Pogz* heterozygote mPFC at P28 (Figure 5) may reflect a homeostatic compensation mechanism that equilibrates levels of these two proteins to minimize the effects on downstream gene expression. The reduction of *Adnp* mRNA was not detected at earlier time points by RNA-seq, which may reflect issues with sensitivity of the assay at those ages, or that the aforementioned homeostatic equilibration mechanism occurs later in development.

POGZ and ADNP are similar proteins, both containing zinc finger domains that bind HP1 proteins, as well as DNA binding domains (ADNP has a homeobox, POGZ has a DDE transposase domain) (Ostapcuk et al., 2018). ADNP syndrome, also known as Helsmoortel-Van Der Aa syndrome, and POGZ syndrome, also known as White-Sutton syndrome, have similar hallmark features: ID with ASD in a subset of patients, developmental delay, and characteristic craniofacial features (Assia Batzir et al., 2020; Van Dijck et al., 2019). The fact that we find these proteins share more than 1,000 genomic targets in the developing telencephalon may lead us to the underlying genes, cell types, and pathways impacted in children with pathogenic variants in POGZ and ADNP.

Another point of convergence of POGZ with ASD risk genes is the enrichment of ASD genes and CHD8 targets in POGZ bound loci in the human mid-fetal cortex (Figure 6). This suggests that POGZ may regulate other ASD risk genes and that POGZ and CHD8 share common downstream targets. Defining the transcription networks co-regulated by POGZ, ADNP, and CHD8 will open the door to elucidating convergent mechanisms that predispose to ASD and DD.

One of the surprising findings of our data is that POGZ C&R bound loci in human and mouse are enriched for SINE and LINE TEs (Figure 7). Like POGZ, ADNP-occupied loci are also enriched for SINE TEs (Kaaij et al., 2019). The 28 and 29bp *de novo* motifs found in POGZ occupied loci are identical to L1 retrotransposon promoter sequences (Sookdeo et al., 2013). The presence of degenerate versions of these TE sequences in euchromatic POGZ C&R peaks suggests that these TEs may have evolved to become neurodevelopmental REs. This finding also suggests that POGZ could be regulating rates of retrotransposition and the resulting somatic mutations. Rett syndrome models show increased L1 retrotransposition in MECP2 knockout cells (Muotri et al., 2010; D’Gama and Walsh 2018). We do not know whether POGZ regulates L1 retrotransposition, although our RNA-seq analysis shows increased RNA levels of L1 LINE genes in *Pogz*^−/−^.

### Limitations of the Study

One of the limitations of our current study is that, although we do find physiological defects in *Pogz*^+/−^ (Cunniff et al., 2020), we did not find strong *Pogz*^+/−^ transcriptional defects. This limits our ability to make conclusions about transcriptional causes of ASD pathology resulting from deleterious heterozygous variants in POGZ. Further study of human *Pogz*^+/−^ patient derived neurons can illuminate the underlying causes of ASD.

## STAR★METHODS

### RESOURCE AVAILABILITY

#### Lead contact

Further information and requests for resources should be directed to the lead contact, John Rubenstein (John.Rubenstein@ucsf.edu).

#### Materials availability

All unique reagents generated in this study are available from the lead contact with a completed Materials Transfer Agreement

#### Data and code availability

- RNA-seq, ATAC-seq, ChIP-seq, and CUT&RUN data generated for this study have been deposited to GEO and dbGAP and are publicly available as of the date of publication. Accession numbers are listed in the key resources table. Any additional information required to access and analyze the data reported in this paper is available from the lead contact upon request.
- This paper does not report original code.
- Any additional information required to reanalyze the data reported in this paper is available from the lead contact upon request.

### EXPERIMENTAL MODEL AND SUBJECT DETAILS

#### Animal models

All procedures and animal care were approved and performed in accordance with National Institutes of Health and the University of California San Francisco Laboratory Animal Research Center (LARC) guidelines, UCSF IACUC approval number AN180174–02. Mice were maintained in social cages on a 12 hr light/dark cycle with free access to food and water; animals were monitored daily for food and water intake. *Pogz*^−/−^*, Pogz*^+/−^, and wild-type littermates were analyzed in this paper. The mice are in the *Mus musculus* C56/Bl6 strain and were analyzed at ages E12.5, E13.5, P0, P10, and P28. For timed pregnancies, noon on the day of the vaginal plug was timed as day 0.5. Animals of both sexes were used in the analyses.

#### Developing human brain samples

Developing human brain samples were obtained with patient consent in strict observance of the legal and institutional ethical regulations. Protocols were approved by the Human Gamete, Embryo, and Stem Cell Research Committee, and the Institutional Review Board at the University of California, San Francisco. Fresh fetal human brain samples were obtained from elective terminations, with no karyotype abnormalities or genetic conditions reported, and transported in freshly made Cerebral Spinal Fluid on ice (CSF). Samples ranged from 17 gw to 18 gw in age and the sex was male. All dissections and experiments were performed within two hours of tissue acquisition. Dissections of the cortical sample acquired included the entire telencephalic wall, from the ventricular zone to the meninges.

#### CRISPR mouse generation

SgRNAs were designed using the guide design tool at crispr.mit.edu. The following sgRNAs in Table S6 were cloned into the px330 vector (Zhang lab) and sgRNAs were generated by *in vitro* transcription using the MEGAshortscript T7 transcription kit (Invitrogen). Both sgRNAs were injected into fertilized mouse oocytes together with Cas9 protein at the Gladstone Transgenic Core facility. Mice were screened for 10 kb deletions using PCR Primers (Table S6) upstream and downstream of the two sgRNA target sites.

### METHOD DETAILS

#### *In situ* hybridization

E13.5 whole head was dissected and postfixed overnight in 4% paraformaldehyde, and transferred to 30% sucrose overnight. Heads were frozen in OCT on dry ice and 20 micron thick cryosections were obtained and stored at −80C. *In situ* hybridization using antisense RNA probes in Table S6 was performed as follows: slides were defrosted at room temperature for twenty minutes. Slides were washed four times, 5 minutes each time, in 1x PBS to removed OCT compound. Slides were fixed in 4% PFA in 1X PBS at room temperature for 10 minutes, and rinsed three times, three minutes each time, in 1x PBS. Slides were treated with Proteinase K (1 μg/ml in 1x PBS) for 17 minutes at room temperature. After Proteinase K digestion, slides were postfixed in 4% PFA at room temperature for 5 minutes, followed by three three-minute washes in 1× PBS. Slides were incubated in acetylation solution (200mL Milli-Q water, 2.66 mL Triethanolamine, 0.35mL 37% HCL, and 0.75 mL acetic anhydride added dropwise) on a shaker for ten minutes, followed by three five-minute washes in 1× PBS. Slides were then incubated for two hours at room temperature in hybridization buffer (140mL 100% formamide, 75 mL 20X SSC pH = 4.5, 300 μL 50 mg/mL yeast tRNA, 3 g SDS, 300 μL 50mg/mL Heparin, final volume 300 μL in dH2O). Antisense probe (Table S6) was diluted to a final concentration of 1 ng/μL in hybridization buffer and 120 μL placed onto the slide under glass coverslips. Slides were placed in a hybridization oven at 72C overnight.

Next, slides were incubated in pre-warmed 5X SSC at 72C for five minutes, and then incubated twice in 0.2X SSC thirty minutes each at 72C. Slides were washed in room temperature 0.2X SSC for five minutes, then incubated in NTT solution (30mL 5M NaCl, 100mL 1M TrisCL (pH = 8.0), 4mL 25% Tween-20 and 916 mL dH2O), and slides were blocked for one hour in 500 μL blocking reagent (500uL 5% Heat Inactivated Sheep Serum in 9.5 mLs 2% blocking reagent in NTT). Blocking reagent was aspirated from sections and 180uL antibody solution was added (2uL anti-Digoxigenin-AP Fab fragment in 10mL blocking solution). Sections were incubated with antibody at 4C overnight. Coverslips were removed the next day in NTT solution, and slides were washed three times in NTT, 30 minutes each wash. Slides were washed in NTTML (24mL 5M NaCL, 40mL filtered 1M TrisCl (pH = 9.5), 3.2 mL 25% Tween-20, 40mL 1M MgCl2, 1mL 1.6M Levamisole, final volume 800mL). BM Purple was added to slides and sections were developed at 37C and were imaged two days later.

#### Immunofluorescence

E13.5 whole head was dissected and postfixed overnight in 4% paraformaldehyde, and transferred to 30% sucrose overnight. Heads were frozen in OCT and 20 micron thick cryosections were obtained. Immunostaining was performed as follows: slides were washed three times in 1X PBS 0.1% Triton at room temperature. Slides were blocked in 1X PBS 0.1% Triton and 4% Donkey Serum at room temperature for one hour. Primary antibodies (Table S6) were added in 1x PBS 0.1% Triton and 4% donkey serum overnight at 4C at 1:1000 dilution. Next day, slides were washed three times in 1x PBS 0.1% Triton, and secondary antibodies (Alexa Fluor) were added at 1:1000 dilution in 1x PBS 0.1% Triton 4% Donkey Serum. Slides were washed three more times in 1X PBS 0.1% Triton and mounted using Vectashield with DAPI.

IF images were obtained using a Zeiss Confocal Microscope at 20X or 63X magnification. Cortical cell numbers were counted in ImageJ using the cell counter plugin to quantify the number of positive cells in the VZ. VZ length was measured by comparing to a 5mm scale image taken using the same microscope. Cortical layer thickness was quantified using ImageJ by comparing the thickness of TBR+ or β-III Tubulin+ layers to a 5mm scale image. Statistical tests were performed in R Studio.

#### Luciferase assay

Primers in Table S6 were used to amplify predicted regulatory elements bound by POGZ from mouse genomic DNA, then cloned into the minimal promoter pGl4.23 luciferase vector (Promega) using SacI and XhoI restriction sites (underlined) in the vector’s multiple cloning site.

Neonatal cortical and basal ganglia tissues were dissected from CD1 mice in cold EBSS, followed by trypsin (Thermo Fisher Scientific 25200056) treatment for 15 minutes at 37°C. Trypsinization was inhibited using 10% FBS containing DMEM. Cells were washed once with DMEM, then resuspended in 10% FBS containing Neuralbasal-A medium (Thermo Fisher Scientific 12348017) with B27 (Thermo Fisher Scientific 17504044). Cell density was quantified using hemocytometer. Cells were plated in poly-D-lysine and laminin coated coverslips (Corning 08–774-385) preloaded in 96 well plates and cultured in 37°C incubator for 14 days. Serum free Neuralbasal-A medium with B27 and Glutamax (Thermo Fisher Scientific 35050061) was used to maintain the cell growth.

Confluent cells were transfected in three 96-well plates with luciferase vectors (candidate enhancer-pGL4.23 or empty vector pGL4.23) and pRL renilla vector. Two days later, cells were lysed and luciferase levels detected using the Promega dual reporter luciferase assay kit. Luciferase levels were normalized to Renilla and averaged across three replicate experiments.

#### Co-immunoprecipitation

Microdissected cortices from wild-type E13.5 mouse embryos or 18 gw human embryos were triturated using P1000 tip 10 times to generate single cell suspension. To isolate nuclei, cells were resuspended in 1mL hypotonic lysis buffer (10mM HEPES pH 7.4, 10mM NaCl, 1.5mM MgCl_2_, 0.5mM DTT, and 1x Roche complete protease inhibitor) per 1E7 cells, held on ice for 10 minutes, and dounced 10–15 times with tight clearance pestle. Nuclei were collected by centrifugation and resuspended in nuclear extraction buffer (20mM HEPES pH 7.4, 300mM NaCl, 25% glycerol, 0.1mM EGTA, 0.02% Igepal, 0.5mM DTT, 250U Benzonase, and 1× Roche complete protease inhibitor) and incubated at 37°C for 15 minutes to digest DNA and RNA. Nuclei were rotated for 30 minutes at 4°C and then centrifuged for 30 minutes at 14,000 × g at 4°C to pellet insoluble fraction. Nuclear extracts were diluted 1:1 with nuclear extraction buffer lacking NaCl for a final concentration of 150mM.

For immunoprecipitations, Dynabeads Protein A (Thermo Fisher, 10001D) were washed twice with IP buffer and added to 5–10μL of antibody suspended in 1% BSA in PBS for two hours at room temperature. Beads were washed twice more with IP buffer and added to nuclear extracts overnight at 4°C. After separation from unbound lysate, beads were washed 5 times with IP buffer and bound proteins were eluted by incubating in 100μL Laemmli Sample Buffer at 70°C for 10 minutes.

#### Western blot

Nuclear extracts were prepared as described above for Co-Immunoprecipitation, from wild-type, heterozygote, and homozygote cortex. 1ug protein was loaded for each genotype in 2x Laemmli Sample Buffer, and POGZ was blotted using the recommended dilution of antibody (1:1000); anti Histone H3 antibody was included for loading control. Relative levels of POGZ protein were measured by pixel intensity analysis of scanned western blot images using ImageJ.

#### MRI

4% PFA-fixed brains were washed twice in 20 mL PBS for a total of 24 hours and imaged in Fluorinert FC−40 (Sigma Aldrich) for null background signal. The imaging was done on a 600 MHz NMR spectrometer (Agilent Technologies Inc.) with imaging gradients and the following parameters: 3D gradient echo, TE/TR 15/75 ms, 8 averages, field of view (FOV) 12.8 mm isotropic, resolution of 50 μm × 50 μm × 100 μm, and a total scan time of 5.5 hours. The acquired images were converted on console to the DICOM format. Volumetric measurements were made using Horos, an open source image viewer.

#### RNA-seq

From each dissection of E13.5 cortex or basal ganglia, RNA was isolated using QIAGEN RNeasy mini columns. RNA was treated with Turbo DNase and inactivated. RNA quality was assessed using Agilent Bioanalyzer RNA Nano kit, and samples with RNA Integrity Number values greater than 9.0 were used for subsequent profiling. RNA-seq libraries were generated using Nugen’s Ovation Mouse RNA-seq kit and amplified for 15 cycles. Libraries were quantified using Agilent Bioanalyzer High Sensitivity DNA kit, and sequenced on Hiseq 2500 using paired end sequencing.

#### ATAC-seq

From each dissection of E13.5 cortex or basal ganglia, intact nuclei were isolated by pipetting up and down twenty times in ice cold 0.5 mL Buffer 1 (300mM sucrose, 60mM KCl, 15mM NaCl, 15mM Tris-HCl, pH 7.5, 5mM MgCl2, 0.1mM EGTA, 1mM DTT, 1.1mM PMSF, Protease inhibitors), and then lysed on ice for 10 minutes after adding 0.5 mL Buffer 2 (300mM sucrose, 60mM KCl, 15mM NaCl, 15mM Tris-HCl, pH 7.5, 5mM MgCl2, 0.1mM EGTA, 0.1% NP-40, 1mM DTT, 1.1mM PMSF, Protease inhibitors). During these ten minutes, nuclei were counted using trypan blue and 50,000 nuclei were spun down at 7,000rpm for ten minutes at 4C. Nuclei were resuspended in 25uL Tagmentation buffer, 22.5 uL Nuclease Free H20, and 2.5 uL Tagmentation Enzyme from Nextera DNA Library Prep Kit, gently mixed, and placed in 37C water bath for thirty minutes. The tagmentation reaction was stopped by MinElute PCR purification and DNA was eluted in 10uL Nuclease Free water. ATAC-seq library generation was performed using Illumina barcode oligos as described, for 8–11 cycles PCR using NEBNext High Fidelity 2x PCR master mix. The number of cycles was empirically determined for each library by qPCR. Libraries were bioanalyzed using Agilent High Sensitivity DNA Kit, pooled together and sequenced on Hiseq 2500 using paired end sequencing.

#### ChIP-seq

From each dissection of E13.5 cortex, intact nuclei were isolated by pipetting up and down twenty times in ice cold 0.5 mL Buffer 1 (300mM sucrose, 60mM KCl, 15mM NaCl, 15mM Tris-HCl, pH 7.5, 5mM MgCl2, 0.1mM EGTA, 1mM DTT, 1.1mM PMSF, 50mM Sodium Butyrate, EDTA-free Protease inhibitors), and then lysed on ice for 10 minutes after adding 0.5 mL Buffer 2 (300mM sucrose, 60mM KCl, 15mM NaCl, 15mM Tris-HCl, pH 7.5, 5mM MgCl2, 0.1mM EGTA, 0.1% NP-40, 1mM DTT, 1.1mM PMSF, 50mM Sodium Butyrate, EDTA-free Protease inhibitors). During this ten minutes, nuclei were counted using trypan blue and 500,000 nuclei were spun down at 7,000rpm for ten minutes at 4C. Nuclei were resuspended in 0.250mL MNase buffer (320mM sucrose, 50mM TrisHCl, pH 7.5, 4mM MgCl2, 1mM CaCl2, 1.1mM PMSF, 50mM Sodium Butyrate) and incubated in a 37C water bath with 2 μl Micrococcal Nuclease enzyme (NEB) for eight minutes. Micrococcal Nuclease digestion was stopped by adding 10 μl 0.5M EDTA, and chromatin was spun down for 10 minutes 10,000rpm 4C. Soluble fraction “S1” supernatant was saved at 4C overnight, and “S2” fraction was dialyzed overnight in 250uL dialysis buffer at 4C (1mm Tris-HCl pH 7.5, 0.2mM EDTA, 0.1mM PMSF, 50mM Sodium Butyrate, Protease Inhibitors). Next day S1 and S2 fractions were combined, 50 μl were saved as input, and Chromatin immunoprecipitation was set up in ChIP buffer: 50mM Tris, pH 7.5, 10mM EDTA, 125 mM NaCl1, 0.1% Tween. 250mM Sodium Butyrate was supplemented for H3K27ac ChIPs. 1 μl of antibody was added to 1mL chromatin in ChIP buffer and incubated overnight at 4C rotating. Protein A and Protein G beads (10 μl for each ChIP) were blocked overnight in 700uL ChIP buffer, 20 uL yeast tRNA (20mg/mL), and 300uL BSA (10mg/mL). Beads were washed three times for five minutes on ice in Wash buffer 1 (50 mM Tris, pH 7.5, 10mM EDTA, 125mM NaCl, 0.1% Tween-20, with protease inhibitors and 5mM sodium butyrate) and three times in Wash buffer 2 (50 mM Tris, pH 7.5, 10mM EDTA, 175mM NaCl, 0.1% NP-40, with protease inhibitors and 5mM sodium butyrate), and ChIP DNA was eluted in elution buffer at 37C and purified by phenol chloroform extraction and ethanol precipitation. Sequencing libraries were made using Nugen Ovation Ultralow V2 kit and quantified by Agilent High Sensitivity DNA kit on the Agilent bioanalyzer.

#### CUT&RUN

From each dissection of E13.5 telencephalon, intact nuclei were isolated using Buffer 1 and Buffer 2 as for ChIP-seq and ATAC-seq above. From each dissection of human cortical tissue, intact nuclei were isolated by douncing in 1mL Buffer 1 with loose pestle and lysing in 1mL Buffer 2. After spinning down nuclei at 7,000rpm for ten minutes at 4C, we methodically followed the protocol for CUT&RUN (Skene, Henikoff, and Henikoff 2018), starting at step 6 “Resuspend in 1mL of wash buffer at RT by gentle pipetting.” Antibodies were used at the following dilutions: POGZ 1:500, ADNP 1:13, Hp1γ 1:500, IgG 1:1000. For the final DNA extraction step, we performed phenol-chloroform extraction and ethanol precipitation. We generated libraries using Nugen’s Ovation Ultralow V2 kit, and amplified libraries for 16 cycles, and bioanalyzed libraries using the Agilent High Sensitivity DNA kit and sequenced libraries on the Hiseq 2500.

### QUANTIFICATION AND STATISTICAL ANAYLYSIS

#### ATAC-seq analysis

We mapped groomed fastq files to the mm9 genome using Bowtie2 default mode (Langmead and Salzberg 2012). Samtools merge (v1.10) was used to merge experimental replicates together before peak calling (Li et al., 2009). For peak calling, we used MACS2 (v2.1.1) *callpeak* (Zhang et al., 2008). We disabled model-based peak calling and local significance testing. We used a fixed fragment extension length of 200bps, and we used a q-value cut-off of 0.05. We used the peak calling results to run MACS2 differential peak calling (bdgdiff) with a min peak length of 150bps. To visualize differences in ATAC-seq signal in wild-type versus *Pogz*^−/−^ on the genome browser, we used DeepTools bamCompare tool, dividing the genome into bins of 50 kb. To measure differences in ATAC-seq signal over gene bodies of *Pogz*^−/−^ cortex DE genes and all unchanged genes as controls, we used DeepTools bigwig-Compare tool, comparing the two genotypes using log2 option, dividing the genome into bins of 50bp. Then we used Bedtools (Quinlan and Hall 2010) intersect function to intersect with bed files of DE gene body coordiantes. Log2(KO/WT) values were plotted in R and differences in the means between up- or downregulated genes and unchanged genes were tested using Student’s t test.

#### ChIP-seq analysis

We mapped groomed fastq files to the mm9 genome using Bowtie2 default mode (Langmead and Salzberg 2012). We used samtools merge (v1.10)(Li et al., 2009) to merge experimental replicates together before peak calling. For peak calling, we used MACS2 (v2.1.1) *callpeak* (Zhang et al., 2008). We disabled model-based peak calling and local significance testing. We used a fixed fragment extension length of 200bps, and we used a q-value cut-off of 0.05. We used the peak calling results to run MACS2 differential peak calling (bdgdiff) with a min peak length of 150bps.

#### Motif analysis

We used Homer to call motifs on our peak sets, using a size window of 200 (Heinz et al., 2010). We used the findMotifsGenome.pl and annotatePeaks.pl tools to acquire sets of enriched motifs for all our experiments and annotations for our peaks. We used MEME-Chip to call *de novo* motifs in our peak sets, using default settings (Bailey et al., 2015). We subsequently used FIMO with default settings to scan POGZ C&R peaks for individual instances of *de novo* motifs (Bailey et al., 2015). From FIMO output we determined if the instance of the motif was degenerate or matched the consensus motif exactly (non-degenerate), and we generated heatmaps of ATAC-seq on those peak sets using DeepTools computeMatrix and plotHeatmap tools (Ramírez et al., 2016).

#### CUT&RUN analysis

We mapped groomed fastq files to the mm9 genome using Bowtie2 default mode (Langmead and Salzberg 2012). Samtools merge (v1.10) was used to merge experimental replicates together before peak calling (Li et al., 2009). For peak calling, we used MACS2 (v2.1.1) *callpeak* (Zhang et al., 2008). We disabled model-based peak calling and local significance testing. We used broad peak settings and a q-value cut-off of 0.05. Bedtools (Quinlan and Hall 2010) was used to determine consensus peaks across replicates and antibodies. Heatmaps of CUT&RUN signal for various antibodies over POGZ C&R peaks were generated using DeepTools computeMatrix and plotHeatmap tools (Ramírez et al., 2016). To plot CUT&RUN signal at consensus POGZ/ADNP/HP1γ peaks proximal to differentially expressed genes, we used Bedtools to intersect consensus peak bed files with bed files of differentially expressed genes (q-value < 0.01) from cortex *Pogz*^−/−^ (Table S2) extended by 100kB upstream and downstream of gene locations. We then used DeepTools computeMatrix and plotHeatmap tools (Ramírez et al., 2016) to plot the distribution of CUT&RUN signal for POGZ, ADNP, and HP1γ at those peaks.

#### RNA-seq analysis

RNA-seq reads were mapped to mm9 using HISAT2 version 2.0.5 (Kim et al., 2019), and reads were counted on mouse genes using htseq version 0.6.1p1 (Anders, Pyl, and Huber 2014). Differentially expressed transcripts were determined from count tables using DESeq2 version 1.14.1, and genes with q-value < 0.05 were included in plots and tables of differentially expressed genes. For RNA-seq analysis of transposable element expression, we mapped reads to the mm9 genome using RNA STAR (Dobin et al., 2013) and counted reads on repeatmasked sequences of the mm9 genome using featureCounts (Liao, Smyth, and Shi 2014). Differentially expressed transcripts were determined from count tables using DESeq2, and genes with q-value < 0.05 were included in figures and supplemental tables. All RNA-seq experiments were run in single batch preparations, as they were done on single litters, so batch correction was not performed.

#### GO Analysis

GO analysis for genomic loci (CUT&RUN, differential ATAC-seq, H3K27ac ChIP-seq peaks) was performed using GREAT version 4.0.4 (McLean et al., 2010) and the default setting for single nearest gene with 1,000 kb for associating genomic regions with genes. GO analysis for DE genes (RNA-seq) was performed using DAVID Bioinformatics Resources 6.8 (Huang, Sherman, and Lempicki 2009). Only GO terms with q-value < 0.05 were reported, after multiple hypothesis correction.

#### Transposable Element Analysis

Performed separately for human (hg19) and mouse (mm9) genomes, each genome was divided into non-overlapping 100bp windows (excluding ENCODE blacklisted regions) and intersected with repeat coordinates from Tandem Repeats Finder (obtained from the UCSC Genome Browser) as well as POGZ peaks (CUT&RUN). This was performed for each repeat family using separate euchromatic and heterochromatic POGZ peaks. The odds ratio and p value was computed from the contingency table of genomic bins overlapping a particular repeat family versus those overlapping a particular set of CUT&RUN peaks. P values were adjusted for multiple testing (Benjamini-Hochberg).

#### Gene set enrichment analysis

We tested if various gene sets were enriched near two different sets of C&R peaks (POGZ and H4K20me3): ASD risk genes, developmental delay risk genes, CHD8 targets, Fragile-X targets (An et al., 2018), lung expressed genes, liver expressed genes, olfactory receptor genes, and random subsets of 512 genes expressed with mean rpkm > 3 in whole brain from Brainspan 16 gw-19 gw samples (Hawrylycz et al., 2012). Enrichment was tested over the space of all protein coding genes, with one vector representing if a gene was in the gene set, and another vector representing if a gene was within 50 kilobases of a statistically significant C&R peak. A 2×2 contingency table was computed from these vectors, and Fisher’s exact test used to compute the odds ratio and p value. The resulting p values from all gene set and C&R target combinations were corrected for multiple testing (Benjamini-Hochberg).

We also tested if there was a statistically significant difference in the proportion of CHD8 target genes proximal to POGZ peaks versus the proportion of whole brain expressed genes proximal to human POGZ C&R peaks peaks. The latter proportion was computed using all whole brain expressed genes (Li et al., 2018), and the empirical p value of the proportion of POGZ-proximal CHD8 target genes was computed using the resampled distribution of POGZ-proximal expressed whole brain gene proportions. This resulted in an empirical p value < 0.001, as the maximum proportion of POGZ-proximal random whole brain genes was 56.9 (mean 53.4), while the proportion of POGZ-proximal CHD8 gene targets was 68.2.

All genes names were converted to their current HUGO names for compatibility. Whole brain genes with mean RPKM > 3 for 16–19 gestational week samples in RNA-seq data from Brainspan were defined as expressed.

## Supplementary Material

supplemental Figures 1-7

supplemental Table 1

supplemental table 2

supplemental Table 4

supplemental table 3

supplemental table 6

supplemental table 5

## Figures and Tables

**KEY RESOURCES TABLE:** 

Reagent or resource	Source	Identifier

Antibodies		

anti-H3K27ac Clone CMA309	Millipore	Cat# 05–1334; RRID:AB_1977244
anti-H4K20me3	Abcam	Cat# ab9053; RRID:AB_306969
anti-POGZ	Abcam	Cat# ab171934
anti-IgG	Stem Cell Technologies	Cat# 60129AV
anti-ADNP	Invitrogen	Cat# PA5–47792; RRID:AB_2576251
rabbit anti-goat IgG	Abcam	Cat# ab6697; RRID:AB_955988
anti-HP1gamma	Abcam	Cat# ab10480; RRID:AB_297219
anti-HP1alpha	Abcam	Cat# ab77256; RRID:AB_1523784
anti-beta III tubulin	Novus	Cat# NB100–1612; RRID:AB_10000548
anti-TBR2	Cell signaling	Cat# 4540S; RRID:AB_1903959

Chemicals, Peptides, and Recombinant Proteins		

B-27 supplement	Thermo Fisher	Cat#12587–010
N2 supplement	Thermo Fisher	Cat#17502–048
pA-MNase	Skene et al., 2018	N/A

Critical Commercial Assays		

MEGAshortscript T7 transcription kit	Thermo Fisher	Cat#AM1354
MinElute PCR Purification Kit	QIAGEN	Cat#28004
RNeasy plus mini kit	QIAGEN	Cat#7413
High Sensitivity DNA Kit	Agilent	Cat#5067–4626
RNA 6000 nano kit	Agilent	Cat#5067–1511
Nextera DNA Library Prep Kit	Illumina	Cat#FC-121–1030
Ovation Ultralow System V2	Nugen	Cat#0344NB-32
NEBNext High Fidelity 2x PCR master mix	New England Biolabs	Cat#M0541L
Micrococcal Nuclease	New England Biolabs	Cat#M0247S
Dual-Luciferase Reporter Assay System	Promega	Cat#E1980
Turbo Dnase	Thermo Fisher	Cat#AM2238

Deposited Data		

RNA-seq data, mouse	this paper	GEO: GSE187010
ATAC-seq data, mouse	this paper	GEO: GSE187010
ChIP-seq data, mouse	this paper	GEO: GSE187010
CUT&RUN data, mouse	this paper	GEO: GSE187010
CUT&RUN data, human	this paper	dbGAP: phs002033.v2.p1
ATAC-seq data, human	Markenscoff-Papadimitriou et al., 2020	GEO: GSE149268
ChIP-seq data, human	Markenscoff-Papadimitriou et al., 2020	GEO: GSE149268
Disease gene sets for gene set enrichment analysis	An et al., 2018	https://science.sciencemag.org/content/362/6420/eaat6576/tab-figures-data
VISTA positive and negative enhancers	Visel et al., 2007	https://enhancer.lbl.gov/
Genes expressed in whole fetal brain	Hawrylycz et al., 2012	https://www.brainspan.org/static/download.html

Experimental Models: Organisms/Strains		

Mouse: POGZ ex1–6 deletion	this paper	N/A

Oligonucleotides		

sgRNA targeting exon 1	GGCGGACACCGACCTGTTTA	N/A
sgRNA targeting exon 6	TGTCACTGTGAAGCGACCTG	N/A
forward sequencing primer, pogz deletion mouse	CCAGGCCAGCCTATTTTACA	N/A
reverse sequencing primer, pogz deletion mouse	CCCACTGGTTCTTTGAGCAG	N/A

Recombinant DNA		

pGL4.23[luc2/minP] Vector	Promega	Cat#E841A
pRL Vector	Promega	Cat#E2261
pX330-U6-Chimeric_BB-CBh-hSpCas9	Addgene	Cat#42230

Software and Algorithms		

bedtools2	Quinlan and Hall, 2010	https://github.com/arq5x/bedtools2
macs2	Zhang et al., 2008	https://github.com/macs3-project/MACS
Homer	Heinz et al., 2010	http://homer.ucsd.edu/homer/
GREAT	McLean et al., 2010	http://great.stanford.edu/public/html/

## Highlights

- POGZ binds enhancers and promoters at euchromatic loci in the developing brain.
- POGZ promotes neuronal gene expression and chromatin accessibility.
- POGZ forms a nuclear complex with ADNP; *Adnp* expression is reduced in *Pogz*^+/−^.
- POGZ bound sites are enriched for other autism risk genes and transposable elements.

## References

[R1] Abelson JF, Kwan KY, O’Roak BJ, Baek DY, Stillman AA, Morgan TM, Mathews CA, Pauls DL, Rasin MR, Gunel M, (2005). Sequence variants in SLITRK1 are associated with Tourette’s syndrome. Science 310, 317–320. 16224024 10.1126/science.1116502

[R2] An J-Y, Lin K, Zhu L, Werling DM, Dong S, Brand H, Wang HZ, Zhao X, Schwartz GB, Collins RL, (2018). Genome-wide de novo risk score implicates promoter variation in autism spectrum disorder. Science 362, eaat6576. 30545852 10.1126/science.aat6576 PMC6432922

[R3] Anders S, Pyl PT, and Huber W (2014). “HTSeq – A Python framework to work with high-throughput sequencing data.”. Cold Spring Harbor Laboratory. 10.1101/002824. PMC4287950 25260700

[R4] Aruga J, and Mikoshiba K (2003). Identification and characterization of Slitrk, a novel neuronal transmembrane protein family controlling neurite outgrowth. Mol. Cell. Neurosci. 24, 117–129. 14550773 10.1016/s1044-7431(03)00129-5

[R5] Assia Batzir N, Posey JE, Song X, Akdemir ZC, Rosenfeld JA, Brown CW, Chen E, Holtrop SG, Mizerik E, Nieto Moreno M, ; Baylor-Hopkins Center for Mendelian Genomics (2020). Phenotypic expansion of POGZ-related intellectual disability syndrome (White-Sutton syndrome). Am. J. Med. Genet. A 182, 38–52. 31782611 10.1002/ajmg.a.61380 PMC7713511

[R6] Bailey TL, Johnson J, Grant CE, and Noble WS (2015). The MEME Suite. Nucleic Acids Res. 43 (W1), W39–49. 25953851 10.1093/nar/gkv416 PMC4489269

[R7] Beaubien F, Raja R, Kennedy TE, Fournier AE, and Cloutier J-F (2016). Slitrk1 is localized to excitatory synapses and promotes their development. Sci. Rep. 6, 27343. 27273464 10.1038/srep27343 PMC4895136

[R8] Canzio D, Larson A, and Narlikar GJ (2014). Mechanisms of functional promiscuity by HP1 proteins. Trends Cell Biol. 24, 377–386. 24618358 10.1016/j.tcb.2014.01.002 PMC4077871

[R9] Cappi C, Oliphant ME, Péter Z, Zai G, Conceição do Rosário M, Sullivan CAW, Gupta AR, Hoffman EJ, Virdee M, Olfson E, (2020). De novo damaging DNA coding mutations are associated with obsessive-compulsive disorder and overlap with Tourette’s disorder and autism. Biol. Psychiatry 87, 1035–1044. 31771860 10.1016/j.biopsych.2019.09.029 PMC7160031

[R10] Cappuccio G, Attanasio S, Alagia M, Mutarelli M, Borzone R, Karali M, Genesio R, Mormile A, Nitsch L, Imperati F, (2019). Microdeletion of pseudogene chr14.232.a affects LRFN5 expression in cells of a patient with autism spectrum disorder. Eur. J. Hum. Genet. 27, 1475–1480. 31152157 10.1038/s41431-019-0430-5 PMC6777536

[R11] Chahrour M, Jung SY, Shaw C, Zhou X, Wong STC, Qin J, and Zoghbi HY (2008). MeCP2, a key contributor to neurological disease, activates and represses transcription. Science 320, 1224–1229. 18511691 10.1126/science.1153252 PMC2443785

[R12] Chang J, Gilman SR, Chiang AH, Sanders SJ, and Vitkup D (2015). Genotype to phenotype relationships in autism spectrum disorders. Nat. Neurosci. 18, 191–198. 25531569 10.1038/nn.3907 PMC4397214

[R13] Cotney J, Muhle RA, Sanders SJ, Liu L, Willsey AJ, Niu W, Liu W, Klei L, Lei J, Yin J, (2015). The autism-associated chromatin modifier CHD8 regulates other autism risk genes during human neurodevelopment. Nat. Commun. 6, 6404. 25752243 10.1038/ncomms7404 PMC4355952

[R14] Cunniff MM, Markenscoff-Papadimitriou E, Ostrowski J, Rubenstein JL, and Sohal VS (2020). Altered hippocampal-prefrontal communication during anxiety-related avoidance in mice deficient for the autism-associated gene *Pogz*. eLife 9, e54835. 33155545 10.7554/eLife.54835 PMC7682992

[R15] D’Gama AM, and Walsh CA (2018). Somatic mosaicism and neurodevelopmental disease. Nat. Neurosci. 21, 1504–1514. 30349109 10.1038/s41593-018-0257-3

[R16] Darnell JC, Van Driesche SJ, Zhang C, Hung KY, Mele A, Fraser CE, Stone EF, Chen C, Fak JJ, Chi SW, (2011). FMRP stalls ribosomal translocation on mRNAs linked to synaptic function and autism. Cell 146, 247–261. 21784246 10.1016/j.cell.2011.06.013 PMC3232425

[R17] De Rubeis S, He X, Goldberg AP, Poultney CS, Samocha K, Cicek AE, Kou Y, Liu L, Fromer M, Walker S, ; DDD Study; Homozygosity Mapping Collaborative for Autism; UK10K Consortium (2014). Synaptic, transcriptional and chromatin genes disrupted in autism. Nature 515, 209–215. 25363760 10.1038/nature13772 PMC4402723

[R18] Deciphering Developmental Disorders Study (2017). Prevalence and architecture of de novo mutations in developmental disorders. Nature. 542, 433–438. 28135719 10.1038/nature21062 PMC6016744

[R19] Deciphering Developmental Disorders Study (2015). Large-scale discovery of novel genetic causes of developmental disorders. Nature. 519, 223–228. 25533962 10.1038/nature14135 PMC5955210

[R20] Dobin A, Davis CA, Schlesinger F, Drenkow J, Zaleski C, Jha S, Batut P, Chaisson M, and Gingeras TR (2013). STAR: ultrafast universal RNA-seq aligner. Bioinformatics 29, 15–21. 23104886 10.1093/bioinformatics/bts635 PMC3530905

[R21] Fang X, Peterson KR, Li Q, and Stamatoyannopoulos G (2003). Locus control regions. In Gene Transfer and Expression in Mammalian Cells, 38, New Conprehensive Biochemistry, Makrides SC, ed. (Elsevier), pp. 397–409.

[R22] Fazel Darbandi S, Robinson Schwartz SE, Qi Q, Catta-Preta R, Pai EL, Mandell JD, Everitt A, Rubin A, Krasnoff RA, Katzman S, (2018). Neonatal Tbr1 dosage controls cortical layer 6 connectivity. Neuron 100, 831–845.e7. 30318412 10.1016/j.neuron.2018.09.027 PMC6250594

[R23] Gompers AL, Su-Feher L, Ellegood J, Copping NA, Riyadh MA, Stradleigh TW, Pride MC, Schaffler MD, Wade AA, Catta-Preta R, (2017). Germline Chd8 haploinsufficiency alters brain development in mouse. Nat. Neurosci. 20, 1062–1073. 28671691 10.1038/nn.4592 PMC6008102

[R24] Gudmundsdottir B, Gudmundsson KO, Klarmann KD, Singh SK, Sun L, Singh S, Du Y, Coppola V, Stockwin L, Nguyen N, (2018). POGZ Is Required for silencing mouse embryonic b-like hemoglobin and human fetal hemoglobin e xpression. Cell Rep. 23, 3236–3248. 29898395 10.1016/j.celrep.2018.05.043 PMC7301966

[R25] Hashimoto R, Nakazawa T, Tsurusaki Y, Yasuda Y, Nagayasu K, Matsumura K, Kawashima H, Yamamori H, Fujimoto M, Ohi K, (2016). Whole-exome sequencing and neurite outgrowth analysis in autism spectrum disorder. J. Hum. Genet. 61, 199–206. 26582266 10.1038/jhg.2015.141 PMC4819764

[R26] Hawrylycz MJ, Lein ES, Guillozet-Bongaarts AL, Shen EH, Ng L, Miller JA, van de Lagemaat LN, Smith KA, Ebbert A, Riley ZL, (2012). An anatomically comprehensive atlas of the adult human brain transcriptome. Nature 489, 391–399. 22996553 10.1038/nature11405 PMC4243026

[R27] Heinz S, Benner C, Spann N, Bertolino E, Lin YC, Laslo P, Cheng JX, Murre C, Singh H, and Glass CK (2010). Simple combinations of lineage-determining transcription factors prime cis-regulatory elements required for macrophage and B cell identities. Mol. Cell 38, 576–589. 20513432 10.1016/j.molcel.2010.05.004 PMC2898526

[R28] Heyne HO, Singh T, Stamberger H, Abou Jamra R, Caglayan H, Craiu D, De Jonghe P, Guerrini R, Helbig KL, Koeleman BPC, ; EuroEPINOMICS RES Consortium (2018). De novo variants in neurodevelopmental disorders with epilepsy. Nat. Genet. 50, 1048–1053. 29942082 10.1038/s41588-018-0143-7

[R29] Huang W, Sherman BT, and Lempicki RA (2009). Systematic and integrative analysis of large gene lists using DAVID bioinformatics resources. Nat. Protoc. 4, 44–57. 19131956 10.1038/nprot.2008.211

[R30] Iossifov I, O’Roak BJ, Sanders SJ, Ronemus M, Krumm N, Levy D, Stessman HA, Witherspoon KT, Vives L, Patterson KE, (2014). The contribution of de novo coding mutations to autism spectrum disorder. Nature 515, 216–221. 25363768 10.1038/nature13908 PMC4313871

[R31] Jung E-M, Moffat JJ, Liu J, Dravid SM, Gurumurthy CB, and Kim W-Y (2017). Arid1b haploinsufficiency disrupts cortical interneuron development and mouse behavior. Nat. Neurosci. 20, 1694–1707. 29184203 10.1038/s41593-017-0013-0 PMC5726525

[R32] Kaaij LJT, Mohn F, van der Weide RH, de Wit E, and Bühler M (2019). The ChAHP complex counteracts chromatin looping at CTCF sites that emerged from SINE expansions in mouse. Cell 178, 1437–1451.e14. 31491387 10.1016/j.cell.2019.08.007

[R33] Kim D, Paggi JM, Park C, Bennett C, and Salzberg SL (2019). Graph-based genome alignment and genotyping with HISAT2 and HISAT-genotype. Nat. Biotechnol. 37, 907–915. 31375807 10.1038/s41587-019-0201-4 PMC7605509

[R34] Langmead B, and Salzberg S (2012). Fast gapped-read alignment with Bowtie 2. Nature Methods. 9, 357–359. 22388286 10.1038/nmeth.1923 PMC3322381

[R35] Li H, Handsaker B, Wysoker A, Fennell T, Ruan J, Homer N, Marth G, Abecasis G, and Durbin R; 1000 Genome Project Data Processing Subgroup (2009). The sequence alignment/map format and SAMtools. Bioinformatics 25, 2078–2079. 19505943 10.1093/bioinformatics/btp352 PMC2723002

[R36] Li M, Santpere G, Imamura Kawasawa Y, Evgrafov OV, Gulden FO, Pochareddy S, Sunkin SM, Li Z, Shin Y, Zhu Y, ; BrainSpan Consortium; PsychENCODE Consortium; PsychENCODE Developmental Subgroup (2018). Integrative functional genomic analysis of human brain development and neuropsychiatric risks. Science 362, eaat7615. 30545854 10.1126/science.aat7615 PMC6413317

[R37] Liao Y, Smyth GK, and Shi W (2014). featureCounts: an efficient general purpose program for assigning sequence reads to genomic features. Bioinformatics 30, 923–930. 24227677 10.1093/bioinformatics/btt656

[R38] Lindtner S, Catta-Preta R, Tian H, Su-Feher L, Price JD, Dickel DE, Greiner V, Silberberg SN, McKinsey GL, McManus MT, (2019). Genomic resolution of DLX-orchestrated transcriptional circuits driving development of forebrain GABAergic neurons. Cell Rep. 28, 2048–2063.e8. 31433982 10.1016/j.celrep.2019.07.022 PMC6750766

[R39] Linhoff MW, Laurén J, Cassidy RM, Dobie FA, Takahashi H, Nygaard HB, Airaksinen MS, Strittmatter SM, and Craig AM (2009). An unbiased expression screen for synaptogenic proteins identifies the LRRTM protein family as synaptic organizers. Neuron 61, 734–749. 19285470 10.1016/j.neuron.2009.01.017 PMC2746109

[R40] Markenscoff-Papadimitriou E, Whalen S, Przytycki P, Thomas R, Binyameen F, Nowakowski TJ, Kriegstein AR, Sanders SJ, State MW, Pollard KS, and Rubenstein JL (2020). A chromatin accessibility atlas of the developing human telencephalon. Cell 182, 754–769.e18. 32610082 10.1016/j.cell.2020.06.002 PMC7415678

[R41] Matsumura K, Nakazawa T, Nagayasu K, Gotoda-Nishimura N, Kasai A, Hayata-Takano A, Shintani N, Yamamori H, Yasuda Y, Hashimoto R, and Hashimoto H (2016). De novo POGZ mutations in sporadic autism disrupt the DNA-binding activity of POGZ. J. Mol. Psychiatry 4, 1. 27103995 10.1186/s40303-016-0016-x PMC4839133

[R42] Matsumura K, Seiriki K, Okada S, Nagase M, Ayabe S, Yamada I, Furuse T, Shibuya H, Yasuda Y, Yamamori H, (2020). Pathogenic POGZ mutation causes impaired cortical development and reversible autism-like phenotypes. Nat. Commun. 11, 859. 32103003 10.1038/s41467-020-14697-z PMC7044294

[R43] McLean CY, Bristor D, Hiller M, Clarke SL, Schaar BT, Lowe CB, Wenger AM, and Bejerano G (2010). GREAT improves functional interpretation of cis-regulatory regions. Nat. Biotechnol. 28, 495–501. 20436461 10.1038/nbt.1630 PMC4840234

[R44] Minc E, Courvalin JC, and Buendia B (2000). HP1gamma associates with euchromatin and heterochromatin in mammalian nuclei and chromosomes. Cytogenet. Cell Genet. 90, 279–284. 11124534 10.1159/000056789

[R45] Muotri AR, Marchetto MCN, Coufal NG, Oefner R, Yeo G, Nakashima K, and Gage FH (2010). L1 retrotransposition in neurons is modulated by MeCP2. Nature 468, 443–446. 21085180 10.1038/nature09544 PMC3059197

[R46] Nozawa R-S, Nagao K, Masuda H-T, Iwasaki O, Hirota T, Nozaki N, Kimura H, and Obuse C (2010). Human POGZ modulates dissociation of HP1α from mitotic chromosome arms through Aurora B activation. Nat. Cell Biol. 12, 719–727. 20562864 10.1038/ncb2075

[R47] O’Roak BJ, Morgan TM, Fishman DO, Saus E, Alonso P, Gratacòs M, Estivill X, Teltsh O, Kohn Y, Kidd KK, (2010). Additional support for the association of SLITRK1 var321 and Tourette syndrome. Mol. Psychiatry 15, 447–450. 20351724 10.1038/mp.2009.105 PMC3292207

[R48] Ostapcuk V, Mohn F, Carl SH, Basters A, Hess D, Iesmantavicius V, Lampersberger L, Flemr M, Pandey A, Thomä NH, (2018). Activity-dependent neuroprotective protein recruits HP1 and CHD4 to control lineage-specifying genes. Nature 557, 739–743. 29795351 10.1038/s41586-018-0153-8

[R49] Parikshak NN, Luo R, Zhang A, Won H, Lowe JK, Chandran V, Horvath S, and Geschwind DH (2013). Integrative functional genomic analyses implicate specific molecular pathways and circuits in autism. Cell 155, 1008–1021. 24267887 10.1016/j.cell.2013.10.031 PMC3934107

[R50] Pinhasov A, Mandel S, Torchinsky A, Giladi E, Pittel Z, Goldsweig AM, Servoss SJ, Brenneman DE, and Gozes I (2003). Activity-dependent neuroprotective protein: a novel gene essential for brain formation. Brain Res. Dev. Brain Res. 144, 83–90. 12888219 10.1016/s0165-3806(03)00162-7

[R51] Quinlan AR, and Hall IM (2010). BEDTools: a flexible suite of utilities for comparing genomic features. Bioinformatics 26, 841–842. 20110278 10.1093/bioinformatics/btq033 PMC2832824

[R52] Rada-Iglesias A, Bajpai R, Swigut T, Brugmann SA, Flynn RA, and Wysocka J (2011). A unique chromatin signature uncovers early developmental enhancers in humans. Nature 470, 279–283. 21160473 10.1038/nature09692 PMC4445674

[R53] Ramírez F, Ryan DP, Grüning B, Bhardwaj V, Kilpert F, Richter AS, Heyne S, Dündar F, and Manke T (2016). deepTools2: a next generation web server for deep-sequencing data analysis. Nucleic Acids Res. 44 (W1), W160–W165. 27079975 10.1093/nar/gkw257 PMC4987876

[R54] Sandberg M, Flandin P, Silberberg S, Su-Feher L, Price JD, Hu JS, Kim C, Visel A, Nord AS, and Rubenstein JLR (2016). Transcriptional networks controlled by NKX2–1 in the development of forebrain GABAergic neurons. Neuron 91, 1260–1275. 27657450 10.1016/j.neuron.2016.08.020 PMC5319854

[R55] Sanders SJ, He X, Willsey AJ, Ercan-Sencicek AG, Samocha KE, Cicek AE, Murtha MT, Bal VH, Bishop SL, Dong S, ; Autism Sequencing Consortium (2015). Insights into autism spectrum disorder genomic architecture and biology from 71 risk loci. Neuron 87, 1215–1233. 26402605 10.1016/j.neuron.2015.09.016 PMC4624267

[R56] Sando R, Jiang X, and Südhof TC (2019). Latrophilin GPCRs direct synapse specificity by coincident binding of FLRTs and teneurins. Science 363, eaav7969. 30792275 10.1126/science.aav7969 PMC6636343

[R57] Satterstrom FK, Kosmicki JA, Wang J, Breen MS, De Rubeis S, An JY, Peng M, Collins R, Grove J, Klei L, ; Autism Sequencing Consortium; iPSYCH-Broad Consortium (2020). Large-scale exome sequencing study implicates both developmental and functional changes in the neurobiology of autism. Cell 180, 568–584.e23. 31981491 10.1016/j.cell.2019.12.036 PMC7250485

[R58] Shmelkov SV, Hormigo A, Jing D, Proenca CC, Bath KG, Milde T, Shmelkov E, (2010). Slitrk5 deficiency impairs corticostriatal circuitry and leads to obsessive-compulsive-like behaviors in mice. Nature Medicine 16, 598–602. 10.1038/nm.2125 PMC2907076 20418887

[R59] Skene PJ, Henikoff JG, and Henikoff S (2018). Targeted in situ genome-wide profiling with high efficiency for low cell numbers. Nat. Protoc. 13, 1006–1019. 29651053 10.1038/nprot.2018.015

[R60] Song M, Giza J, Proenca CC, Jing D, Elliott M, Dincheva I, Shmelkov SV, Kim J, Schreiner R, Huang SH, (2015). Slitrk5 mediates BDNF-dependent TrkB receptor trafficking and signaling. Dev. Cell 33, 690–702. 26004511 10.1016/j.devcel.2015.04.009 PMC4784688

[R61] Sookdeo A, Hepp CM, McClure MA, and Boissinot S (2013). Revisiting the evolution of mouse LINE-1 in the genomic era. Mob. DNA 4, 3. 10.1186/1759-8753-4-3 PMC3600994 23286374

[R62] Stessman HAF, Willemsen MH, Fenckova M, Penn O, Hoischen A, Xiong B, Wang T, Hoekzema K, Vives L, Vogel I, (2016). Disruption of POGZ is associated with intellectual disability and autism spectrum disorders. Am. J. Hum. Genet. 98, 541–552. 26942287 10.1016/j.ajhg.2016.02.004 PMC4890241

[R63] Suliman-Lavie R, Title B, Cohen Y, Hamada N, Tal M, Tal N, Monderer-Rothkoff G, Gudmundsdottir B, Gudmundsson KO, Keller JR, (2020). Pogz deficiency leads to transcription dysregulation and impaired cerebellar activity underlying autism-like behavior in mice. Nat. Commun. 11, 5836. 33203851 10.1038/s41467-020-19577-0 PMC7673123

[R64] Vakoc CR, Mandat SA, Olenchock BA, and Blobel GA (2005). Histone H3 lysine 9 methylation and HP1gamma are associated with transcription elongation through mammalian chromatin. Mol. Cell 19, 381–391. 16061184 10.1016/j.molcel.2005.06.011

[R65] Van Dijck A, Vulto-van Silfhout AT, Cappuyns E, van der Werf IM, Mancini GM, Tzschach A, Bernier R, Gozes I, Eichler EE, Romano C, ; ADNP Consortium (2019). Clinical presentation of a complex neurodevelopmental disorder caused by Mutations in ADNP. Biol. Psychiatry 85, 287–297. 29724491 10.1016/j.biopsych.2018.02.1173 PMC6139063

[R66] Visel A, Minovitsky S, Dubchak I, and Pennacchio LA (2007). VISTA Enhancer Browser–a database of tissue-specific human enhancers. Nucleic Acids Res. 35, D88–D92. 17130149 10.1093/nar/gkl822 PMC1716724

[R67] Willsey AJ, Sanders SJ, Li M, Dong S, Tebbenkamp AT, Muhle RA, Reilly SK, Lin L, Fertuzinhos S, Miller JA, (2013). Coexpression networks implicate human midfetal deep cortical projection neurons in the pathogenesis of autism. Cell 155, 997–1007. 24267886 10.1016/j.cell.2013.10.020 PMC3995413

[R68] Zhang Y, Liu T, Meyer CA, Eeckhoute J, Johnson DS, Bernstein BE, Nusbaum C, Myers RM, Brown M, Li W, and Liu XS (2008). Model-based analysis of ChIP-Seq (MACS). Genome Biol. 9, R137. 18798982 10.1186/gb-2008-9-9-r137 PMC2592715

[R69] Zhang D, Ma X, Sun W, Cui P, and Lu Z (2015). Down-regulated FSTL5 promotes cell proliferation and survival by affecting Wnt/β-catenin signaling in hepatocellular carcinoma. Int. J. Clin. Exp. Pathol 8, 3386–3394. 26045876 PMC4440185

[R70] Zhao W, Tan J, Zhu T, Ou J, Li Y, Shen L, Wu H, Han L, Liu Y, Jia X, (2019). Rare inherited missense variants of POGZ associate with autism risk and disrupt neuronal development. Journal of Genetics and Genomics. 46, 247–257. 31196716 10.1016/j.jgg.2019.04.002

