# Supplementary material for: Autism risk gene POGZ promotes chromatin accessibility and expression of clustered synaptic genes
Source: Cell Rep. Author manuscript; Available in PMC 2022 Sep 26. (PMC9512081; doi:10.1016/j.celrep.2021.110089)
Supplement: supplemental Figures 1-7 [file NIHMS1835097-supplement-supplemental_Figures_1-7.pdf]

Cell Reports, Volume *37*

Supplemental information

Autism risk gene POGZ promotes chromatin accessibility and expression of clustered synaptic genes

Eirene Markenscoff-Papadimitriou, Fadya Binyameen, Sean Whalen, James Price, Kenneth Lim, Athena R. Ypsilanti, Rinaldo Catta-Preta, Emily Ling-Lin Pai, Xin Mu, Duan Xu, Katherine S. Pollard, Alex S. Nord, Matthew W. State, and John L. Rubenstein

Figure S1

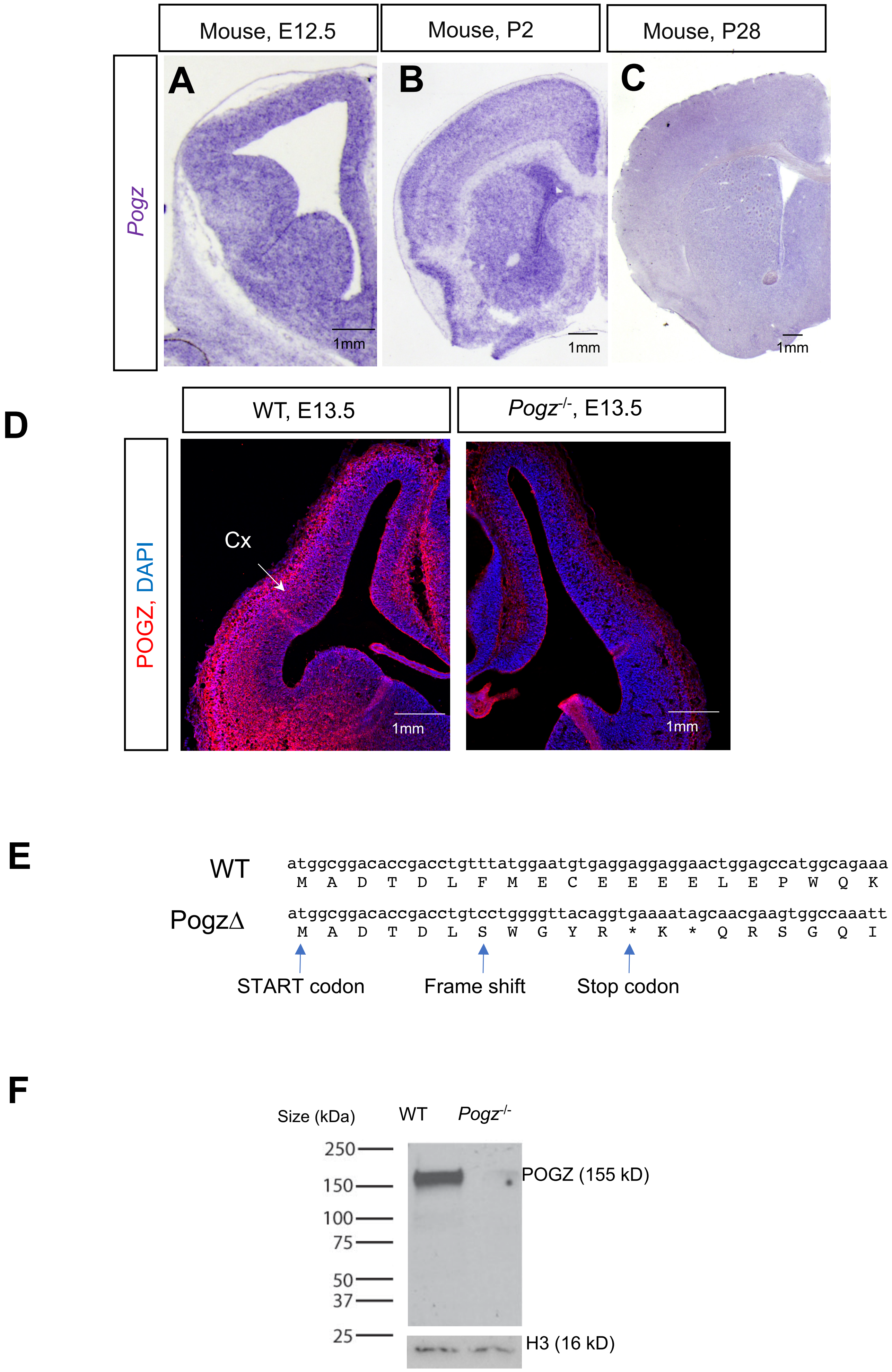

Mouse, E12.5
Mouse, P2
Mouse, P28
A
B
C
Pogz
1mm
1mm
1mm
D
WT, E13.5
Pogz-/-, E13.5
POGZ, DAPI
Cx
1mm
1mm
E
WT
atggcggacaccgacctgtttatggaatgtgaggaggaggaactggagccatggcagaaa
M A D T D L F M E C E E E E L E P W Q K
PogzΔ
atggcggacaccgacctgtcctggggttacaggtgaaaatagcaacgaagtggccaaatt
M A D T D L S W G Y R * K * Q R S G Q I
START codon
Frame shift
Stop codon
F
Size (kDa)
WT
Pogz-/-
250
150
100
75
50
37
25
POGZ (155 kD)
H3 (16 kD)

Figure S1) Generation of POGZ constitutive knockout, Related to Figure 1

S1A-C**)** ISH with antisense *Pogz* probe of wildtype E12.5 (A), postnatal day 2 (B), and postnatal day 28 mouse forebrain (C).

S1D)Anti-POGZ immunostaining (red) and DAPI (blue) in E13.5 wildtype and *Pogz*$^{-/-}$ telencephalon sections. POGZ expression in basal ganglia (BG) and cortex (Cx) is indicated.

S1E) Sanger sequencing of wildtype and *Pogz*$^{-/-}$ alleles. Premature stop codon is indicated in the *Pogz*$^{-/-}$ allele.

S1F) Western blot using anti-POGZ antibody in nuclear extracts from wildtype and *Pogz*$^{-/-}$ mouse cortex, E13.5. Histone H3 loading control.

Figure S2

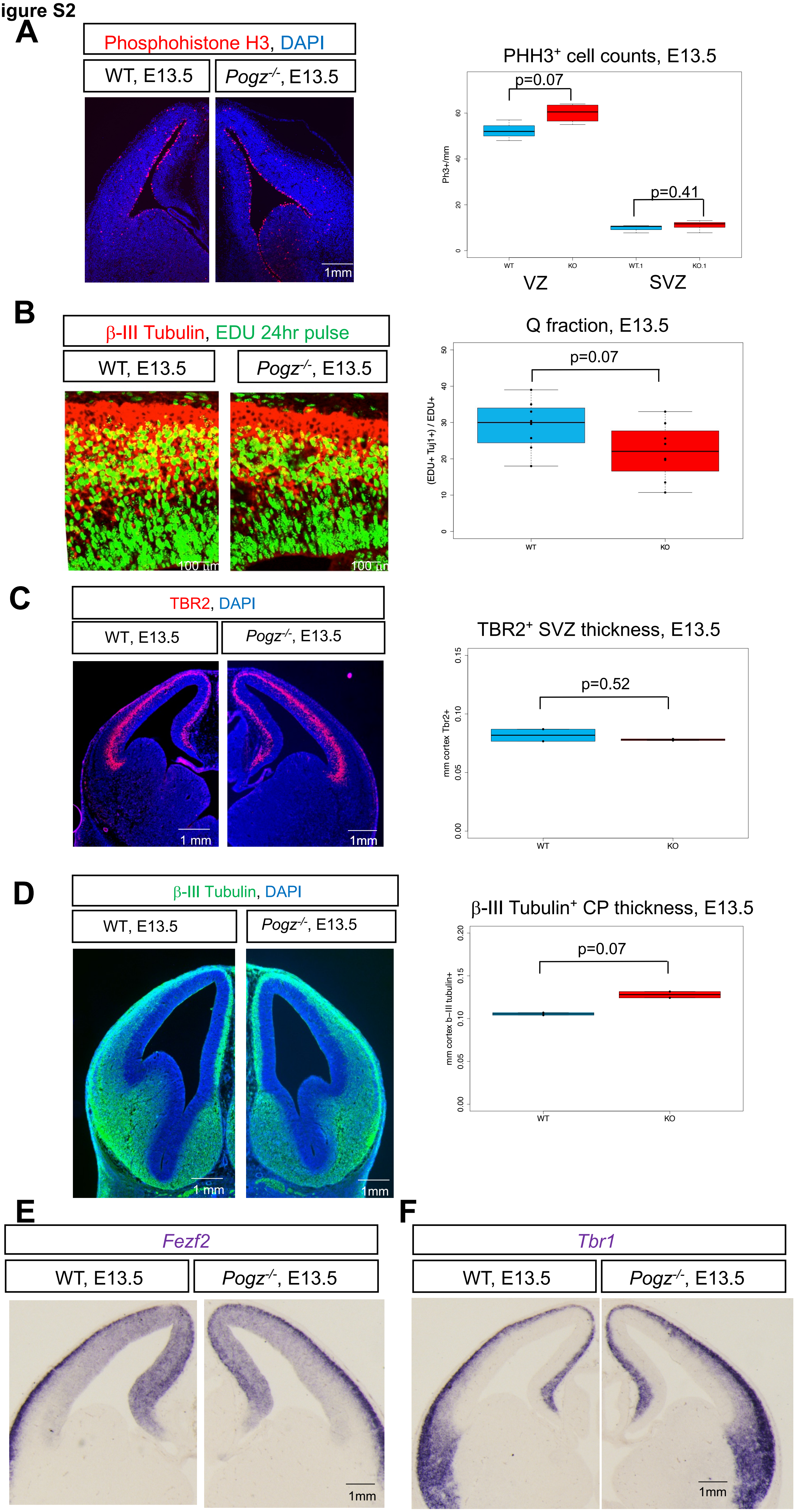
A
Phosphohistone H3, DAPI
WT, E13.5
Pogz-/-, E13.5
1mm
PHH3+ cell counts, E13.5
p=0.07
p=0.41
Ph3+/mm
0
20
40
60
WT
KO
WT.1
KO.1
VZ
SVZ
B
β-III Tubulin, EDU 24hr pulse
WT, E13.5
Pogz-/-, E13.5
Q fraction, E13.5
p=0.07
(EDU+ Tuj1+) / EDU+
0
10
20
30
40
50
WT
KO
C
TBR2, DAPI
WT, E13.5
Pogz-/-, E13.5
1 mm
1mm
TBR2+ SVZ thickness, E13.5
p=0.52
mm cortex Tbr2+
0.00
0.05
0.10
0.15
WT
KO
D
β-III Tubulin, DAPI
WT, E13.5
Pogz-/-, E13.5
1 mm
1mm
β-III Tubulin+ CP thickness, E13.5
p=0.07
mm cortex b−III tubulin+
0.00
0.05
0.10
0.15
0.20
WT
KO
E
Fezf2
WT, E13.5
Pogz-/-, E13.5
1mm
F
Tbr1
WT, E13.5
Pogz-/-, E13.5
1mm

Figure S2) No cortical neurogenesis phenotypes observed at E13.5 *Pogz*$^{-/-}$, Related to Figure 2

S2A) Anti-phosphohistone H3 immunofluorescence (Red) and DAPI (Blue) in E13.5 wildtype and *Pogz*$^{-/-}$, and quantification of positive cells in the cortex ventricular zone (VZ) and subventricular zone (SVZ), n=4.

S2B) 24hr EdU labeling at E12.5 and E13.5 quantification of percent EdU+ cortical cells (green) that are co-labeled with neuronal marker β-III Tubulin (red), n=8.

S2C) Anti-TBR2 immunofluorescence (Red) and DAPI (Blue) in E13.5 wildtype and *Pogz*$^{-/-}$, and quantification of SVZ layer thickness, n=4.

S2D) Anti-β-III Tubulin (Tuj1) immunofluorescence (Green) and DAPI (Blue) in E13.5 wildtype and *Pogz* $^{-/-}$, and quantification of cortical plate (CP) thickness, n=4.

S2E) Layer 5 neuron marker gene *Fezf2* ISH (purple) of E13.5 wildtype and *Pogz*$^{-/-}$.

S2F) Layer 6 neuron marker gene *Tbr1* ISH (purple) of E13.5 wildtype and *Pogz*$^{-/-}$.

Figure S3

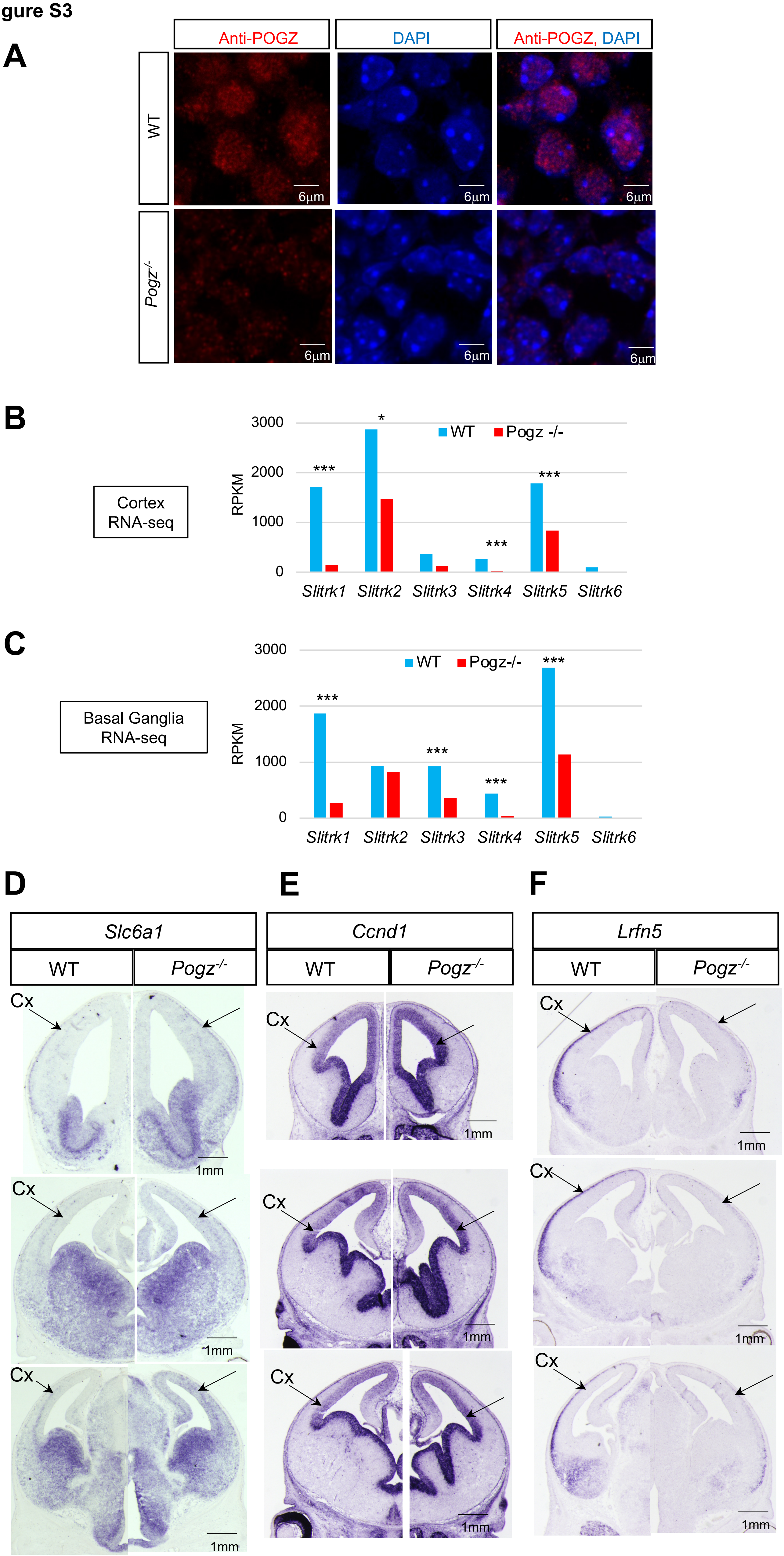

A
Anti-POGZ
DAPI
Anti-POGZ, DAPI
WT
Pogz-/-
6μm
B
Cortex RNA-seq
3000
2000
1000
0
RPKM
WT
Pogz -/-
***
*
***
***
Slitrk1
Slitrk2
Slitrk3
Slitrk4
Slitrk5
Slitrk6
C
Basal Ganglia RNA-seq
3000
2000
1000
0
RPKM
WT
Pogz-/-
***
***
***
***
Slitrk1
Slitrk2
Slitrk3
Slitrk4
Slitrk5
Slitrk6
D
Slc6a1
E
Ccnd1
F
Lrfn5
WT
Pogz-/-
Cx
1mm

Figure S3) RNA-seq analysis of E13.5 *Pogz*$^{-/-}$, Related to Figure 2

S3A) Anti-POGZ immunostaining (red) and DAPI (blue) in E13.5 wildtype and *Pogz*$^{-/-}$ basal ganglia at 63x magnification.

S3B-C) Normalized reads across RNA-seq replicates for wildtype and *Pogz*$^{-/-}$ in E13.5 cortex and basal ganglia, n=3. DE *Slitrk* genes are indicated (***q-value <0.001 , *q-value <0.05).

S3D-F) Coronal sections from rostral (top) to caudal (bottom) of E13.5 wildtype and *Pogz*$^{-/-}$. ISH of genes up-regulated in *Pogz*$^{-/-}$ at E13.5: *Slc6a1* (D), *Ccnd1*(E), and down-regulated gene *Lrfn5*(F).

Figure S4

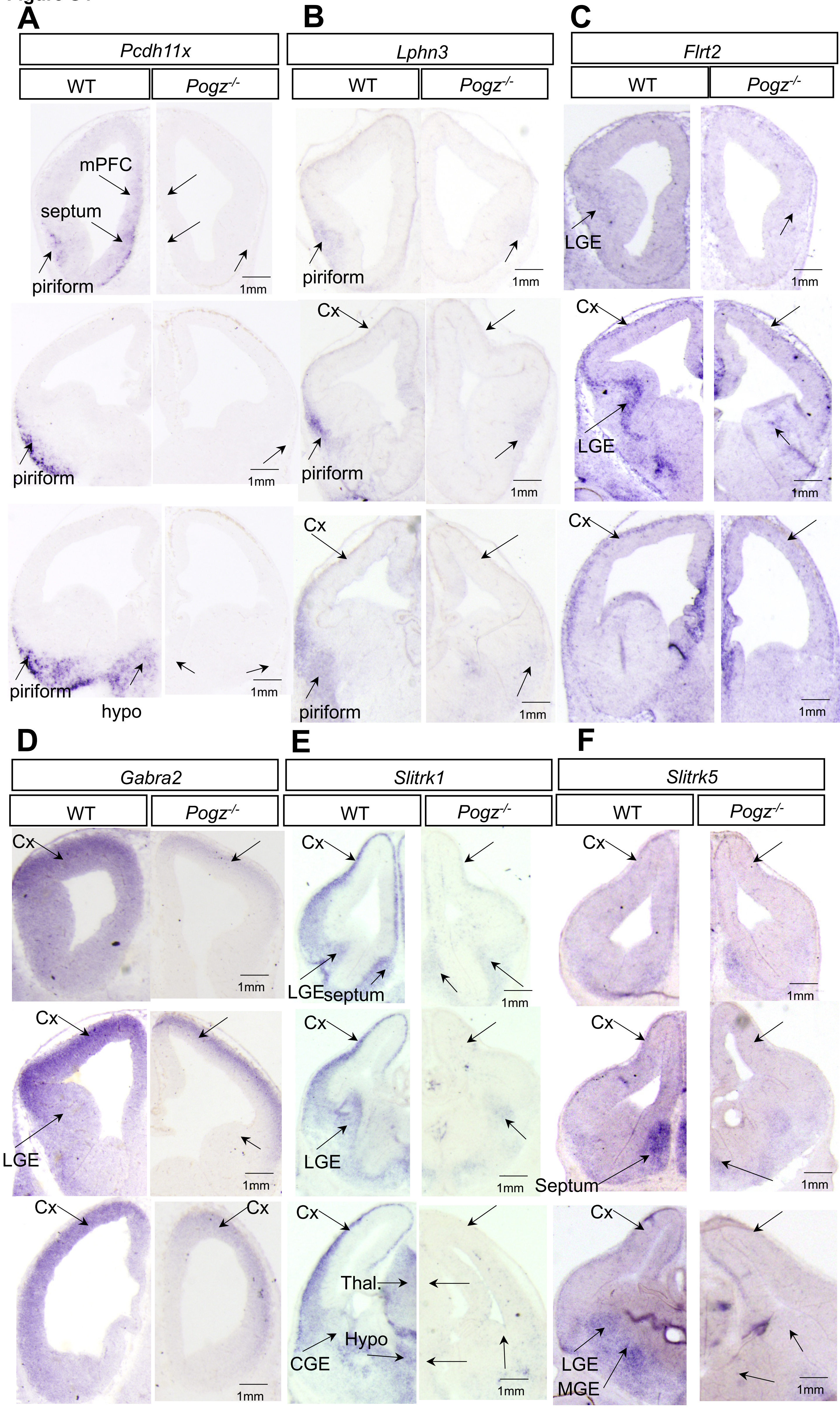
A
Pcdh11x
WT
Pogz-/-
mPFC
septum
piriform
1mm
hypo
B
Lphn3
Cx
C
Flrt2
LGE
D
Gabra2
E
Slitrk1
Thal.
Hypo
CGE
F
Slitrk5
Septum
MGE

Figure S4) ***In situ*** **hybridization validation of** ***Pogz-/-*** **down-regulated genes, Related to Figure 2**

S4A-F) Coronal sections from rostral (top) to caudal (bottom) of E13.5 wildtype and *Pogz*$^{-/-}$ telencephalon. ISH of genes downregulated in *Pogz*$^{-/-}$ at E13.5: *Pcdh11x* (A), *Lphn3* (B), *Flrt2* (C), *Gabra2* (D), *Slitrk1* (E), *Slitrk5* (F).

Figure S5

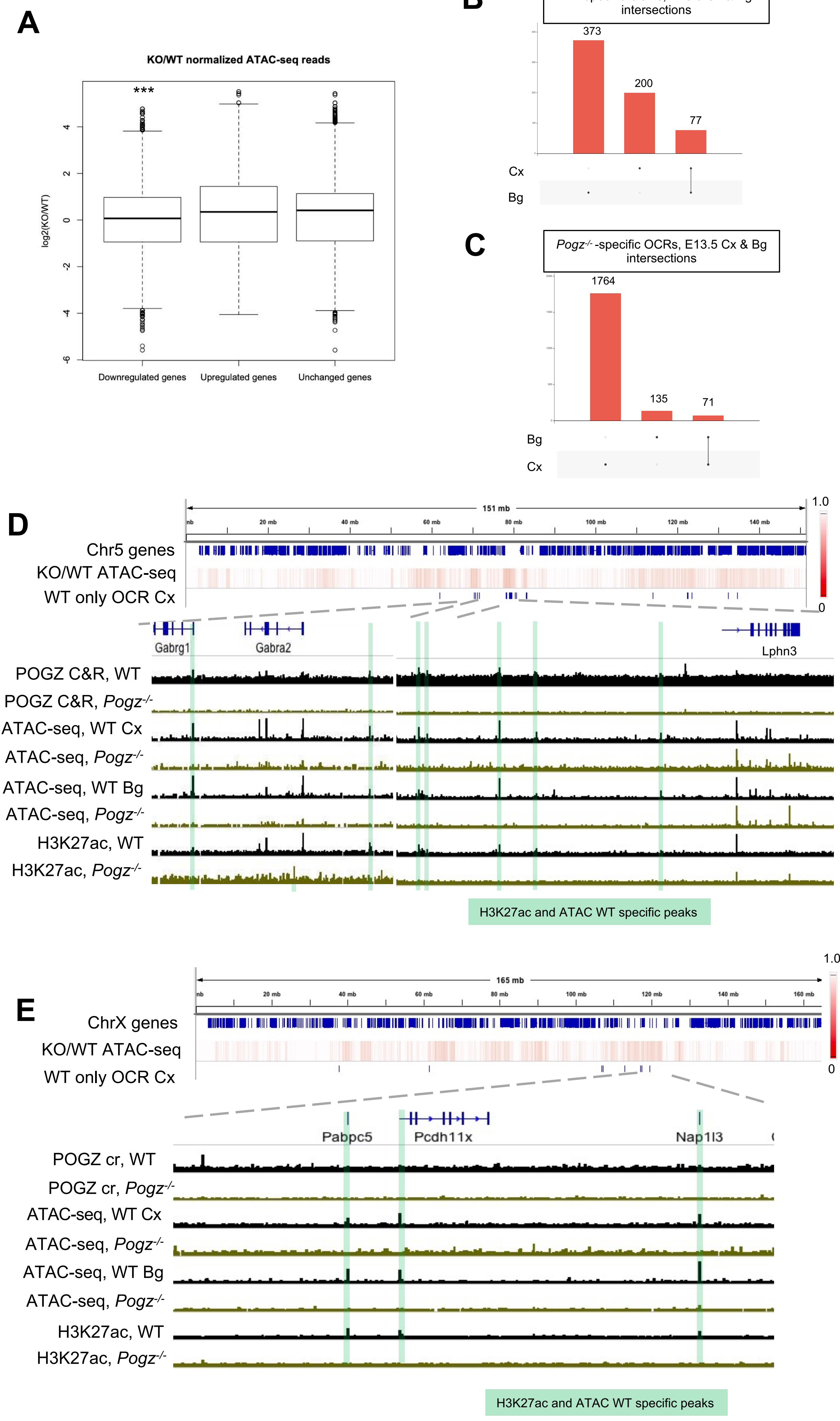

A
KO/WT normalized ATAC-seq reads
***
log2(KO/WT)
4
2
0
-2
-4
-6
Downregulated genes
Upregulated genes
Unchanged genes
B
WT-specific OCRs, E13.5 Cx & Bg intersections
373
200
77
Cx
Bg
C
Pogz-/- -specific OCRs, E13.5 Cx & Bg intersections
1764
135
71
Bg
Cx
D
151 mb
20 mb
40 mb
60 mb
80 mb
100 mb
120 mb
140 mb
1.0
0
Chr5 genes
KO/WT ATAC-seq
WT only OCR Cx
Gabrg1
Gabra2
Lphn3
POGZ C&R, WT
POGZ C&R, Pogz-/-
ATAC-seq, WT Cx
ATAC-seq, Pogz-/-
ATAC-seq, WT Bg
ATAC-seq, Pogz-/-
H3K27ac, WT
H3K27ac, Pogz-/-
H3K27ac and ATAC WT specific peaks
E
165 mb
20 mb
40 mb
60 mb
80 mb
100 mb
120 mb
140 mb
160 mb
1.0
0
ChrX genes
KO/WT ATAC-seq
WT only OCR Cx
Pabpc5
Pcdh11x
Nap1l3
POGZ cr, WT
POGZ cr, Pogz-/-
ATAC-seq, WT Cx
ATAC-seq, Pogz-/-
ATAC-seq, WT Bg
ATAC-seq, Pogz-/-
H3K27ac, WT
H3K27ac, Pogz-/-
H3K27ac and ATAC WT specific peaks

Figure S5) Localized changes in chromatin accessibility at DE gene loci in *Pogz*$^{-/-}$, Related to Figure 3

S5A) Boxplots of log2(KO/WT) normalized ATAC-seq reads overlapping DE and unchanged genes, each data point is a 50bp segment of the genome.

S5B-C) Up-Set plots of intersections between wildtype-specific OCRs in cortex and basal ganglia (B) and *Pogz*$^{-/-}$ specific OCRs in cortex and basal ganglia (C).

S5D) Genome browser view of all genes on mouse chromosome 5. Heatmap of ATAC-seq reads in *Pogz*$^{-/-}$ cortex normalized to wildtype in 10 kb genomic bins. Blue hashes are wildtype-specific OCRs in cortex (Cx) and basal ganglia (Bg). Highlighted are POGZ occupied loci that are wildtype specific OCRs and have wildtype specific enrichment of H3K27ac. Tracks are sequencing reads from individual C&R, ATAC-seq and ChIP-seq experiments at E13.5.

S5E) Genome browser view of all genes on the mouse X chromosome. Heatmap of ATAC-seq reads in *Pogz*$^{-/-}$ cortex normalized to wildtype in 10 kb genomic bins. Blue hashes are wildtype-specific OCRs in cortex (Cx) and basal ganglia (Bg). Highlighted are POGZ occupied loci that have both wildtype specific OCRs and H3K27ac peaks. Tracks are sequencing reads from individual C&R, ATAC-seq and ChIP-seq experiments at E13.5.

Figure S6

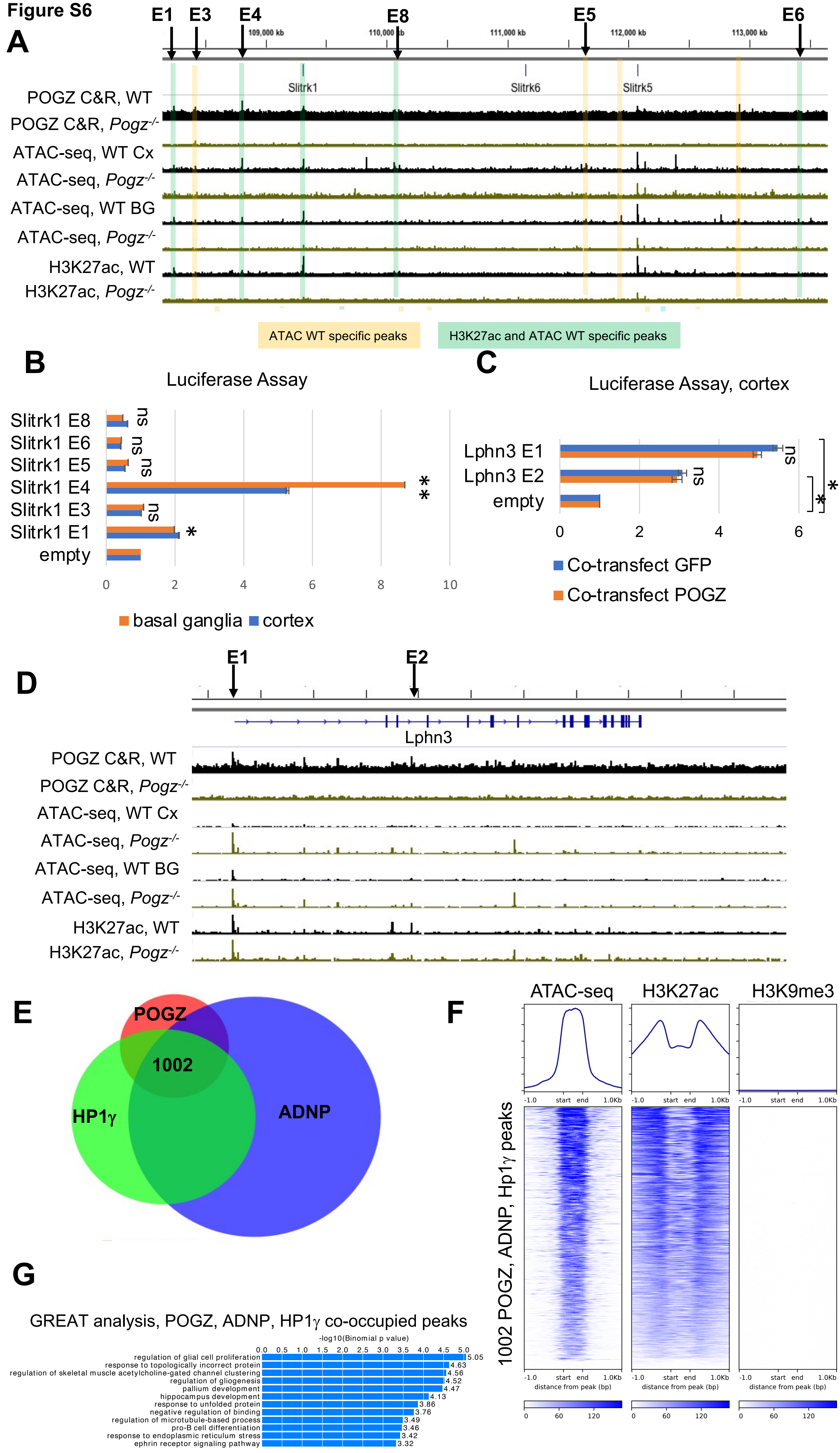

A
E1
E3
E4
E8
E5
E6
109,000 kb
110,000 kb
111,000 kb
112,000 kb
113,000 kb
Slitrk1
Slitrk6
Slitrk5
POGZ C&R, WT
POGZ C&R, Pogz-/-
ATAC-seq, WT Cx
ATAC-seq, Pogz-/-
ATAC-seq, WT BG
ATAC-seq, Pogz-/-
H3K27ac, WT
H3K27ac, Pogz-/-
ATAC WT specific peaks
H3K27ac and ATAC WT specific peaks
B
Luciferase Assay
Slitrk1 E8
Slitrk1 E6
Slitrk1 E5
Slitrk1 E4
Slitrk1 E3
Slitrk1 E1
empty
ns
ns
ns
**
ns
*
0
2
4
6
8
10
basal ganglia
cortex
C
Luciferase Assay, cortex
Lphn3 E1
Lphn3 E2
empty
ns
ns
*
*
0
2
4
6
Co-transfect GFP
Co-transfect POGZ
D
E1
E2
Lphn3
POGZ C&R, WT
POGZ C&R, Pogz-/-
ATAC-seq, WT Cx
ATAC-seq, Pogz-/-
ATAC-seq, WT BG
ATAC-seq, Pogz-/-
H3K27ac, WT
H3K27ac, Pogz-/-
E
POGZ
1002
HP1γ
ADNP
F
ATAC-seq
H3K27ac
H3K9me3
-1.0
start
end
1.0Kb
1002 POGZ, ADNP, Hp1γ peaks
distance from peak (bp)
0
60
120
G
GREAT analysis, POGZ, ADNP, HP1γ co-occupied peaks
-log10(Binomial p value)
0.0
0.5
1.0
1.5
2.0
2.5
3.0
3.5
4.0
4.5
5.0
regulation of glial cell proliferation
5.05
response to topologically incorrect protein
4.63
regulation of skeletal muscle acetylcholine-gated channel clustering
4.56
regulation of gliogenesis
4.52
pallium development
4.47
hippocampus development
4.13
response to unfolded protein
3.86
negative regulation of binding
3.76
regulation of microtubule-based process
3.49
pro-B cell differentiation
3.46
response to endoplasmic reticulum stress
3.42
ephrin receptor signaling pathway
3.32

Figure S6) Pogz bound loci are transcriptional enhancers, and POGZ co-occupies euchromatic loci with ADNP, Related to Figures 3 and 4

S6A) POGZ occupied loci in the *Slitrk1* and *Slitrk5* locus that were tested by luciferase assay (indicated with arrows).

S6B) Mean firefly luciferase levels in primary cortical and basal ganglia cultures normalized to Renilla, normalized to empty vector, for each enhancer candidate in the *Slitrk1* and *Slitrk5* locus.

S6C) POGZ occupied loci in the *Lphn3* locus that were tested by luciferase assay.

S6D) Mean firefly luciferase levels in primary cortical cultures normalized to Renilla, normalized to empty vector, for each enhancer candidate, n=2. Cells were co-transfected with GFP or POGZ expression plasmids.

S6E) Venn Diagram of C&R peak overlaps from ADNP, HP1γ, and POGZ consensus peaks.

S6F) Heat map of ATAC-seq, H3K27ac ChIP-seq, and H3K9me3 ChIP-seq signal across 1002 loci co-occupied by POGZ, ADNP, and HP1γ in E13.5 telencephalon.

S6G) GO analysis of nearest genes to loci co-occupied by POGZ, ADNP, and HP1γ. Significant (q-value<0.05, Benjamini-Hochberg multiple test correction) GO terms listed.

Figure S7

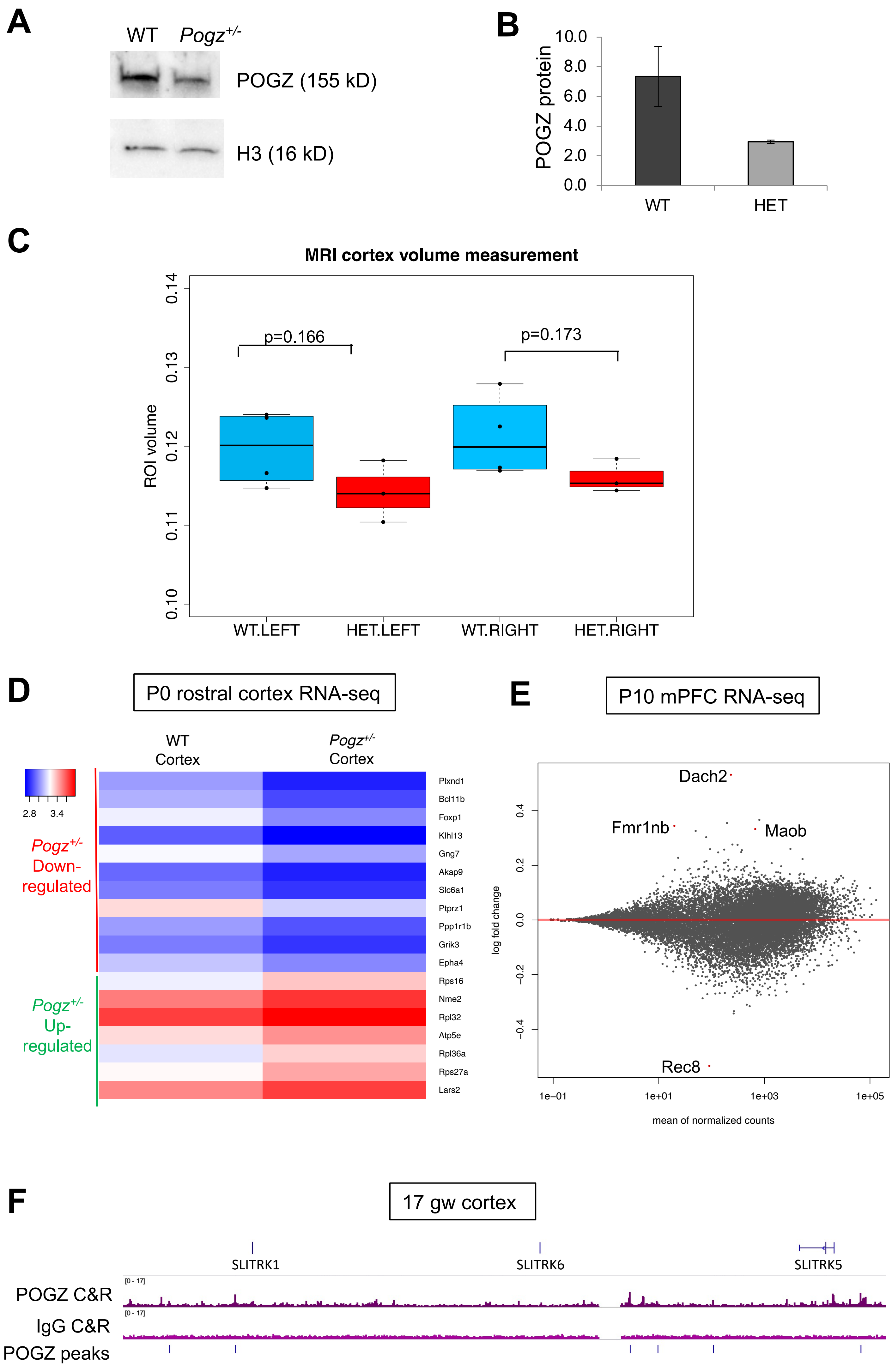
A
WT
Pogz+/-
POGZ (155 kD)
H3 (16 kD)
B
POGZ protein
10.0
8.0
6.0
4.0
2.0
0.0
WT
HET
C
MRI cortex volume measurement
ROI volume
0.14
0.13
0.12
0.11
0.10
p=0.166
p=0.173
WT.LEFT
HET.LEFT
WT.RIGHT
HET.RIGHT
D
P0 rostral cortex RNA-seq
WT Cortex
Pogz+/- Cortex
2.8
3.4
Pogz+/- Down-regulated
Pogz+/- Up-regulated
Plxnd1
Bcl11b
Foxp1
Klhl13
Gng7
Akap9
Slc6a1
Ptprz1
Ppp1r1b
Grik3
Epha4
Rps16
Nme2
Rpl32
Atp5e
Rpl36a
Rps27a
Lars2
E
P10 mPFC RNA-seq
Dach2
Fmr1nb
Maob
Rec8
log fold change
0.4
0.2
0.0
−0.2
−0.4
1e−01
1e+01
1e+03
1e+05
mean of normalized counts
F
17 gw cortex
SLITRK1
SLITRK6
SLITRK5
POGZ C&R
IgG C&R
POGZ peaks
[0 - 17]
[0 - 17]

Figure S7) Analysis of Pogz heterozygote mice, Related to Figure 5

S7A) Western blot using anti-POGZ antibody in nuclear extracts from P2 cortex of *Pogz*$^{+/-}$ and wildtype. Histone H3 loading control.

S7B) Quantification of anti-POGZ signal from Western blots from P28 *Pogz*$^{+/-}$ and wildtype, normalized to loading control, n=5.

S7C) MRI quantification of left and right hemisphere cortex volume from P28 *Pogz*$^{+/-}$ and wildtype mice, n=3.

S7D) Heat map of DE genes in *Pogz*$^{+/-}$ rostral cortex at P0, RNA-seq analysis. Scale is log10 of the average normalized RNA-seq reads for each gene, n=3.

S7E) MA plot showing DE genes in *Pogz*$^{+/-}$ mPFC at P10, RNA-seq analysis. Significant DE genes (q-value <0.05) are indicated in red dots, n=3.

S7F) POGZ and IgG C&R sequencing tracks from human fetal cortex (17 gw) at the SLITRK1 and SLITRK5 locus.
